# Disruption of Notch signaling by KGF induces a developmental pause in thymocytes

**DOI:** 10.3389/fimmu.2025.1675823

**Published:** 2025-11-21

**Authors:** Ruifeng Teng, Francis A. Flomerfelt, Ping Xue, Anjali Chandroth, Constance Tom Noguchi, Nikolaos Svoronos, Ronald E. Gress, Naomi Taylor

**Affiliations:** 1 Experimental Transplantation and Immunology Branch, Center for Cancer Research, National Cancer Institute, National Institutes of Health, Bethesda, MD, United States; 2 Laboratory of Pathology, Center for Cancer Research, National Cancer Institute, National Institutes of Health, Bethesda, MD, United States; 3 Pediatric Oncology Branch, Center for Cancer Research, National Cancer Institute, National Institutes of Health, Bethesda, MD, United States; 4 Molecular Medicine Branch, National Institute of Diabetes and Digestive and Kidney Diseases, National Institutes of Health, Bethesda, MD, United States

**Keywords:** Thymus, NOTCH1, KGF, FOXN1, Wnt/β-catenin, DLL4, β-Selection checkpoint, T cell reconstitution

## Abstract

Keratinocyte growth factor (KGF) has been proposed as a therapeutic adjuvant to enhance T cell immune reconstitution, particularly following stem cell transplantation. Here, we demonstrate that the long-term KGF-induced increase in thymic cellularity and thymocyte differentiation is preceded by a transient developmental block prior to the β-selection checkpoint, observed as early as day 2 following KGF treatment. This early block is characterized by an increased expansion of uncommitted thymocytes and is driven by KGF-induced alterations in both cortical and medullary thymic epithelial cells (TECs). KGF suppresses Wnt/β-catenin signaling by downregulating distinct Wnt ligands in cTECs and mTECs, leading to reduced expression of FOXN1, a master regulator of TEC differentiation. Consequently, expression of *Foxn1*-dependent genes, including *Dll4*, a key Notch ligand required for early thymocyte development, is diminished. These findings reveal a novel mechanism of KGF action: an initial disruption of TEC-mediated signaling that transiently impairs early thymocyte differentiation, followed by enhanced proliferation and long-term thymic recovery.

## 1 Introduction

Thymopoiesis—the development of T cells from early progenitors—is orchestrated within the thymus by a specialized stromal microenvironment composed primarily of mesenchymal cells and thymic epithelial cells (TECs). TECs are subdivided into cortical (cTECs) and medullary (mTECs) subsets, which coordinate the stage-specific progression of thymocytes from multipotent early thymic progenitors (ETPs) to mature CD4^+^ and CD8^+^ single-positive T cells (1, 2). ETPs are the most primary lymphoid/T-cell progenitors in thymus and are categorized as c-Kit^high^ thymocytes within the CD4^−^CD8^−^ double-negative-1 (DN1) subset (3). ETPs progress to the following DN2a stage upon activation of IL2Rα/CD25 expression and concurrent upregulation of T lineage regulatory genes, *Gata-3* and *Tcf-7* (coding for TCF1) expressions. Subsequent progression from DN2a to DN2b occurs when the expression of key T lineage regulatory gene, *Bcl11b*, is initiated and the cell surface expression of both c-Kit and CD44 are downregulated (4–7). Together with GATA-3 and TCF-1, BCL11B induces the activation of pre-T-cell-receptor α (*Ptcra*) and the arrangement of T-cell-receptor β gene (*Trb*), leading to thymocyte progression from the DN2b to DN3a stage. The subsequent assembly of invariant pre-TCRα and TCRβ chain into the pre-TCR complex enables DN3a thymocytes to undergo β-selection, a critical checkpoint in thymocyte development, allowing progression to the DN3b stage and beyond.

Commitment to the T cell lineage and progression through β-selection is driven by transcriptional and signaling networks, with a critical role for the Notch signaling pathway. In particular, sustained Notch1 signaling via Delta-like ligand 4 (DLL4), expressed predominantly on cTECs, is required to maintain expression of lineage-defining genes such as *Bcl11b* (8–12). *Dll4* expression in TECs is transcriptionally regulated by the forkhead box protein FOXN1, a master regulator of TEC development and identity (13, 14). Loss of *Foxn1* leads to diminished DLL4 expression, resulting in thymic hypoplasia and a block in T-lineage commitment (14–16), however, restoration of DLL4 expression together with chemokines CCl25 and CXCL12 in the thymic epithelium reestablished intra-thymic T lineage differentiation in *Foxn1*-deficient embryos (15), highlighting a essential role of DLL4 in orchestrating epithelial–thymocyte crosstalk.

Several studies have shown Wnt/β-catenin signaling as playing a critical role in the regulation of *Foxn1* gene expression and thymic epithelium development during thymic organogenesis and in post-natal thymus (17–19). Excessive Wnt signaling in TECs induced by transgenic expression of a stabilized form of β-catenin resulted in the repression of *Foxn1* gene expression and defective thymic epithelial development. In adult mice, transgenic expression of DKK1, a specific Wnt/β-catenin signaling inhibitor, induced by doxycycline led to repression of *Foxn1* gene expression in TECs (20). In line with the observations from these *in vivo* studies, analyses on TEC cell lines also revealed the expression of endogenous *Foxn1* gene was regulated by Wnt signaling through the β-catenin dependent pathway (21). In addition, the evolutionarily conserved KGF receptor (KGFR)-dependent signaling pathway has also been demonstrated to regulate thymic epithelial development during thymic organogenesis (22, 23). Expression of KGFR (FGFR2IIIb) is restricted to the epithelial cells of a variety of tissues including thymus. KGF (FGF7) and fibroblast growth factor 10 (FGF10) secreted by fibroblast cells in thymic stroma are the main ligands of KGFR in thymus. KGFR transcription starts to be detectable in TECs at around E12, after the initiation of *Foxn1* gene expression and the establishment of thymic epithelium have already occurred. Thus, genetic ablation of KGFR in fetal thymus did not affect the expression of *Foxn1* gene. However, thymic epithelial growth was arrested at E12-E12.5, suggesting KGFR-dependent signaling regulates the proliferation of committed fetal TECs and the expansion of embryonic thymus (23). This effect in promoting proliferation in TECs was recapitulated in adults where KGF administration induced epithelial cell proliferation and associated thymocyte cellularity expansion *in vivo* (24, 25). Despite the potential to enhance thymopoiesis and improve immune reconstitution in preclinical models, clinical trials using KGF in lymphopenic patients have failed to demonstrate consistent benefit in enhancing thymopoiesis or T cell recovery (26, 27). These discrepancies underscore a critical gap in our understanding of how KGF modulates TEC function and the thymic microenvironment.

Here, we investigated the alteration in thymopoiesis and TEC function on day 2 (36–42 hours) following last dose of KGF treatment. KGF induced a rapid expansion of the epithelial compartment but paradoxically led to reduced thymic mass and total thymocyte cellularity. This early impairment was associated with downregulation of *Foxn1* and its transcriptional targets, including *Dll4*, in TECs, resulting in suppression of Notch signaling and diminished *Bcl11b* expression in thymocytes, arresting development at the β-selection checkpoint. Mechanistically, KGF downregulated *Foxn1* through inhibition of *Wnt4* and canonical Wnt/β-catenin signaling, with activation of the MAPK/ERK pathway.

## 2 Materials and methods

### 2.1 Mice

C57BL/6Ncr mice were purchased from Charles River Laboratories. *Rag*2^-/-^ mice (Rag2KO) were purchased from The Jackson Laboratory (Bar Harbor, MN, USA) and maintained by homozygous breeding at NCI-Frederick, MD. *Rag^-/-^Il7^-/-^* (DKO) mice were obtained from Dr. Scott K. Durum (Laboratory of Molecular Immunoregulation, CCR, NCI, NIH, Frederick, MD). Experiments were conducted using 7- to 8-wk-old mice. Euthanasia was performed before tissue collection by compressed CO_2_ gas followed by cervical dislocation. Animal care was provided following NIH Animal Use and Care guidelines and experiments were performed following protocols approved by the NIH Committee.

NCI-Frederick is accredited by AAALAC International and adheres to the Public Health Service Policy for the Care and Use of Laboratory Animals. Animal care was provided in accordance with the procedures outlined in the Guide for Care and Use of Laboratory Animals (National Research Council; 1996; National Academy Press; Washington, D.C.).

### 2.2 KGF treatment in mice

KGF (Palifermin) was dissolved in PBS and administrated via intraperitoneal injection at a dose of 5mg/kg once daily for three consecutive days. Control mice received an equivalent volume of PBS. Thymi were collected at day 2 following the final KGF injection to assess early-phase effects, and at 3 and 6 weeks post-treatment to evaluate later phases.

### 2.3 Preparation of thymocytes for flow cytometry assays

Thymi were collected with the thymic capsule intact to avoid tissue damage. Thymus mass was recorded. To release the thymic parenchyma, the capsule was gently grasped at one edge and torn open, creating an initial breach using fine, sharp-tipped dissecting forceps. Using the tips of the forceps, the capsule membrane was torn into pieces until it was completely fragmented, ensuring full exposure and release of the underlying thymic tissue and associated cells. To release thymocytes from stromal tissue, thymic fragments were progressively disrupted by repeated pipetting in 5 mL of cold PBS until no stromal tissue was visible and only the capsule remained. An additional 15 mL of cold PBS was then added to bring the total volume to 20 mL. The cell suspension was filtered through a 40 μm strainer to obtain a single-cell suspension. Cell concentration was measured using the Nexcelom Cellometer Auto T4. Live and dead cells were distinguished by 0.4% trypan blue staining (Lonza).

For flow cytometry, thymocytes were pre-incubated with mouse BD Fc Block™ (anti-mouse CD16/CD32 mAb, clone 2.4G2) at 1.0 µg per 10^6^ cells in 100 µL staining buffer for 10 minutes, Cells were then stained with fluorophore-conjugated antibodies against cell surface markers (a list of all the markers are presented in **Supplementary Table 1**) and analyzed using The BD LSR Fortessa™ Cell Analyzer. Early thymocyte progenitors (ETPs) were defined as c-Kit^high^ thymocytes within the DN1 (Lin^-^CD44^high^CD25^-^) subset. Other subsets within CD4^-^CD8^-^ double negative (DN) development stage were classified as follow: DN2a (Lin^-^c-Kit^high^CD44^high^CD25^+^), DN2b (Lin^-^c-Kit^low^CD44^low^CD25^+^), DN3a (Lin^-^c-Kit^-^CD44^-^CD25^+^CD28^-^), DN3b (Lin^-^c-Kit^-^CD44^-^CD25^+^CD28^+^), DN3c (Lin^-^c-Kit^-^CD44^-^CD25^low^CD28^+^) and DN4 (Lin^-^c-Kit^-^CD44^-^CD25^-^).

For staining of the mouse pre-TCRα chain, cells were first incubated with a primary monoclonal antibody, then washed and incubated with a biotinylated secondary antibody (anti-mouse IgG1, clone A85-1), followed by incubation with PE-conjugated streptavidin.

DN2a, DN2b, and DN3a thymocytes were sorted on a BD FACSAria™ III Cell Sorter following surface marker staining. Throughout all procedures, thymus and cell samples were kept on ice. All antibodies used were commercially available monoclonal antibodies widely validated for murine immune cell characterization.

### 2.4 Preparation of thymic epithelial cells for flow cytometry assays

Individual thymi were collected and trimmed on ice while preserving the integrity of the thymic capsule. Each intact thymus was transferred to a 5 mL round-bottom polystyrene FACS tube pre-filled with 1 mL of enzymatic digestion solution containing collagenase from *Vibrio alginolyticus* (Roche, 10269638001; 1 mg/mL), dispase from *Bacillus polymyxa* (Roche, 10269638001; 1 mg/mL), and DNase-I (Roche, 11284932001; 0.1 mg/mL) in PBS (Ca^2+^/Mg^2+^-free). The thymic capsule was gently disrupted by pipetting using a wide-bore pipette tip and incubated at 37 °C for 45 minutes with intermittent pipetting (15 times every 15 minutes). After the second round of pipetting, once visible tissue debris had settled to the bottom, the supernatant was collected and transferred to a 50 mL centrifuge tube containing ice-cold stopping buffer (PBS supplemented with 2 mM EDTA and 2% bovine serum). An additional 1 mL of fresh enzymatic digestion solution was added to the FACS tube to continue digestion of the remaining tissue. This process was repeated two more times. After the third digestion step, the entire suspension was transferred to the collection tube and combined with stopping buffer. The resulting cell suspension was filtered through a 100 μm nylon mesh and washed twice with sorting buffer (PBS supplemented with 2 mM EDTA and 2% bovine serum) at 400 × g. A sample taken during the second wash was stained with trypan blue and used to assess viability and determine total cell concentration. After cell counting, staining for thymic epithelial cells (TECs) was performed. EpCAM^−^ mesenchymal cells were classified as Ly51^+^ or Ly51^−^ based on Ly51 surface marker expression. EpCAM^+^ TECs were further subdivided into two major subsets: UEA-1^+^/Ly51^−^ medullary TECs (mTECs) and UEA-1^−^/Ly51^+^ cortical TECs (cTECs). MHC-II staining was applied for the determination of immature TEC progenitor (MHC-II^low^) and mature TECs (MHC-II^high^).

For flow cytometric analysis, 5 × 10^6^ cells per sample were pre-incubated with anti-mouse CD16/CD32 monoclonal antibody (BD Pharmingen, clone 2.4G2), then stained with fluorescein-labeled Ulex Europaeus Agglutinin I (UEA-1; Vector Laboratories) and antibodies against CD45 (BioLegend, clone 30-F11), EpCAM (BioLegend, clone G8.8), Ly51 (BioLegend, clone 6C3), and MHC-II (clone M5/114.15.2). For flow cytometric sorting of TECs, all cells from each individual thymus were stained and sorted accordingly.

### 2.5 Intracellular staining for flow cytometry

Intracellular staining for FOXN1 was performed using anti-FOXN1 mouse IgG2b antibody (clone 2/41), kindly provided by Dr. Hans-Reimer Rodewald (German Cancer Center, Germany) (28). Cells were first stained with a Live/Dead fixable violet dye (Life Technologies) to exclude non-viable cells, followed by incubation with anti-mouse CD16/CD32 monoclonal antibody (BD Pharmingen, clone 2.4G2) to block Fc receptors. After an initial wash with FACS-buffer, cells were stained for surface markers for 20 minutes. Cells were then washed again with FACS buffer and resuspended in 100 µL of Foxp3 Fixation/Permeabilization buffer (eBioscience 00-5123–43 and 00-5223-56) and incubated on ice for 30 minutes. Following fixation, cells were washed with permeabilization buffer (eBioscience, 00-8333-56), then stained for 20 minutes with either anti-FOXN1 or a mouse IgG2b isotype control (BD Pharmingen, clone MPC-11; 1:200). After two additional washes with permeabilization buffer, cells were stained with Alexa Flour 647-conjugated anti-mouse IgG secondary antibody (Invitrogen, 1:800). Throughout the entire staining procedure, cells were kept on ice and protected from light to preserve fluorochrome integrity.

For intracellular staining of the mouse TCRβ chain, thymocytes were fixed, permeabilized, and then stained with PE-conjugated anti-mouse TCRβ monoclonal antibody (clone H57-579).

### 2.6 BrdU incorporation assay

On day 2 following the final dose of KGF or PBS treatment, BrdU (BD 559619, 2mg/mouse) was administrated via intraperitoneal injection 2 hours prior to euthanasia and thymus collection. Thymocytes were isolated and stained as described in the previous section. BrdU incorporation was assessed using the BD Pharmingen FITC BrdU Flow Kit (BD Pharmingen, 559619), following the manufacturer’s instructions.

### 2.7 TE-71 cell culture and transfection

TE-71 cells were maintained in RPM1–1640 medium supplemented with 10% fetal bovine serum (FBS), 5x10–^5^ M 2-mercaptoethanol, 100U/ml of penicillin and l00μg/ml of streptomycin. Cells were passaged at a 1:4 ratio using trypsin/EDTA when they reached approximately 80% confluence. To enhance KGFR expression, 1x10^6^ TE-71 cells were transfected with 2 μg of the pCMV6 vector containing the mouse *Fgfr2* open reading frame (transcript variant 2, encoding FGFR2IIIb; OriGene, MC221076), using the Amaxa Cell Line Nucleofector Kit V (Lonza, VCA-1003) and program T-030. At 24 hours post-transfection, the medium was replaced with fresh medium containing 0.4mg/ml of G418 (Gibco, Life Technology). After 2 weeks of selection in culture with G418, transfected cells expanded clonally and reached 60–70% confluence. Quantitative gene expression analysis confirmed a >100-fold increase in *Kgfr* expression compared to non-transfected cells.

Both non-transfected and transfected TE-71 cells (TE-71*^kgfr^*) were washed three times with warm PBS, then cultured in fresh medium in which 10% FBS was replaced with 20% KnockOut™ Serum Replacement (Gibco). Cells were incubated in this KnockOut medium for 24 hours prior to experimental treatment.

To investigate the effect of KGF, TE-71*^kgfr^* cells were cultured in KnockOut medium supplemented with KGF (Palifermin, 100 ng/ml), either alone or in combination with LiCl (30 mM; Sigma) or the ERK phosphorylation inhibitor U0126 (10 μM, Cell Signaling Technology). The PI3K inhibitor LY294002 was used at a final concentration of 10 μM (Cell Signaling Technology).

### 2.8 Thymic fragment culture

Thymi were collected from 8-week-old C57BL/6 mice and cut into square blocks measuring approximately 2 × 2 × 2 mm. The thymic blocks were placed on the surface of transwell cell culture inserts (pore size: 0.4 µm; Corning) in 6-well plates, with four fragments per well. The tissues were cultured in DMEM supplemented with 10% fetal calf serum (FCS), 1× nonessential amino acids, 10 mM HEPES, 0.05 mM β-mercaptoethanol, 4 mM L-glutamine, and 100 IU/ml penicillin/100μg/ml streptomycin. After 24 hours, the culture medium was aspirated and the wells were washed three times with warm PBS. Then KnockOut medium, DMEM containing the same supplements, except that 10% FCS was replaced with 20% KnockOut™ Serum Replacement (Gibco, ThermoFisher Sientific), was added. The thymic fragments were cultured for an additional 24 hours in this KnockOut medium before being subjected to experimental treatments.

To investigate the effect of KGF, thymic fragments were cultured in KnockOut medium supplemented with KGF (Palifermin, 100 ng/ml), either alone or in combination with the ERK phosphorylation inhibitor U0126 (10 μM).

### 2.9 Mouse IL-7 ELISA

Mouse IL-7 concentrations were measured using the Mouse IL-7 Quantikine ELISA Kit (M7000, R&D systems). Thymic lobes were placed individually into Eppendorf tubes and cut into small pieces on ice immediately after harvesting from mice. Subsequently, 60μl of cold PBS (4°C) was added to each tube. The samples were vortexed for 15 seconds and then centrifuged at 21,000×g for 5 minutes. A total of 50μl of the supernatant, containing intrathymic free IL-7, was used to measure IL-7 concentrations according to the manufacturer’s instructions.

### 2.10 Quantitative Real-Time PCR (qRT-PCR)

Total RNA was extracted from TECs, thymocytes, TE-71 cells, and thymic fragments, and then reverse-transcribed into cDNA using High-Capacity cDNA Reverse Transcription Kit (Thermo Fisher Scientific). qRT-PCR was performed using *TaqMa*n Gene Expression Assays on a QuantStudio™ 7 system (Thermo Fisher Scientific). Ct values for both the internal control gene (Hprt) and target genes (listed in **Supplementary Table 2**) were obtained, and relative gene expression levels were calculated using the 2^−ΔΔCt^ method (Livak and Schmittgen, 2001).

To prevent amplification of contaminating genomic DNA, a DNA elimination step was included during RNA extraction. Additionally, all *TaqMan* probes used (**Supplementary Table 2**) were designed to span exon-exon junctions. Gene expression assays were performed in three independent experiments.

### 2.11 Immunoblotting for AKT and ERK phosphorylation

After 6 hours of incubation with KGF, TE-71*^kgfr^* cells were washed twice with cold PBS and lysed in RIPA buffer (89901, Thermo Fisher Scientific) supplemented with Halt Protease Inhibitor Cocktail (78430, Thermo Fisher Scientific) and Halt Phosphatase Inhibitor Cocktail (78420, Thermo Fisher Scientific). Cell lysates were kept on ice for 30 minutes, with mechanical disruption performed at the 15-minute mark using an ultrasonic processor (Sonicator XL2020) at a frequency of 20 kHz for 10 seconds. Protein concentrations were determined using the Pierce™ BCA Protein Assay kit (23225, Thermo Fisher Scientific).

Following denaturation in NuPAGE LDS sample buffer, equal amounts of protein were subjected to NuPAGE Bis-Tris protein gels and transferred to nitrocellulose membranes, which were then probed with the following rabbit monoclonal antibodies (Cell Signaling Technology): anti-Akt (clone C67E7, #4691S), anti-phospho-Akt (Ser473, clone D9E, #4060S), anti-ERK1/2 (clone 137F5, #4695S), and anti-phospho-ERK1/2 (Thr202/Tyr204, clone D13.14.4E, #4370S). GAPDH was used as an internal control and detected using anti-GAPDH (clone 14C10, #2118L). HRP-conjugated goat anti-rabbit IgG secondary antibody was used for signal detection. Protein bands were visualized by chemiluminescence, and band intensities were quantified using ImageJ software.

### 2.12 Immunofluorescent staining

For KGFR staining, frozen thymus sections were washed three times in cold PBS (4°C) for 5 minutes each, then fixed in cold methanol (4°C) for 15 minutes, followed by additional PBS washes. Tissues were then incubated in blocking solution (5% goat serum in PBS) for 1 hour at room temperature. After blocking, tissue sections were incubated overnight at 4°C with a combination of primary antibodies: anti-mouse FGFR2IIIb rat IgG2a (clone 133730, #MAB7161, R&D Systems) together with either anti-mouse Keratin 5 rabbit IgG (clone AF138, #PRB-160P, Covance) or anti-mouse Keratin 8 rabbit IgG (clone EP1628Y, #ab53280, Abcam). Rat IgG2a (R&D Systems) and rabbit IgG (I-1000, Vector Laboratories) were used as isotype controls for the primary antibodies. For the following secondary staining, thymus sections were incubated with AF488-conjugated goat anti-rat IgG (H+L) (#4416, Cell Signaling Technology) and AF594-conjugated goat anti-rabbit IgG (H+L), F(ab’)_2_ fragment antibodies (#8889, Cell Signaling Technology) at a 1:500 dilution in blocking solution for 1 hour at room temperature. Nuclei were counterstained with DAPI. Images were captured on a Zeiss LSM 790 confocal microscope.

For staining Keratin 5 and Keratin 8 in TE-71 cells, cells were fixed in cold ethanol (4 °C) for 30 minutes, washed three times in cold PBS for 5 minutes each, and blocked in 5% goat serum with 0.1% Tween-20 in PBS for 1 hour at room temperature. Cells were then incubated overnight at 4 °C with primary antibodies: rabbit anti-mouse Keratin 5 IgG (clone AF138, #PRB-160P, Covance) and rat anti-mouse Keratin 8 (clone TROMA-I, Hybridoma Bank), diluted 1:100 in blocking solution with gentle shaking. After PBS washes, cells were incubated with AF488-conjugated goat anti-rat IgG (#4416, Cell Signaling Technology) and AF594-conjugated goat anti-rabbit IgG (#8889, Cell Signaling Technology) for 1 hour at room temperature. Nuclei were counterstained with DAPI. Confocal images were acquired using a Zeiss LSM 790 confocal microscope.

### 2.13 Statistical analysis

Statistical significance between two groups was determined using unpaired two-tailed t-tests. One-way ANOVA was applied for comparison of 3 and more groups and p-values were determined by Tukey’s multiple comparisons test. All data are presented as means ± SD. A p-value of ≤ 0.05 was considered significant for all analyses.

## 3 Results

### 3.1 KGF induces transient decrease in total thymocyte number despite concurrently increasing cell number of DN1-DN3a subsets at day 2 post treatment

**Figure 1:**
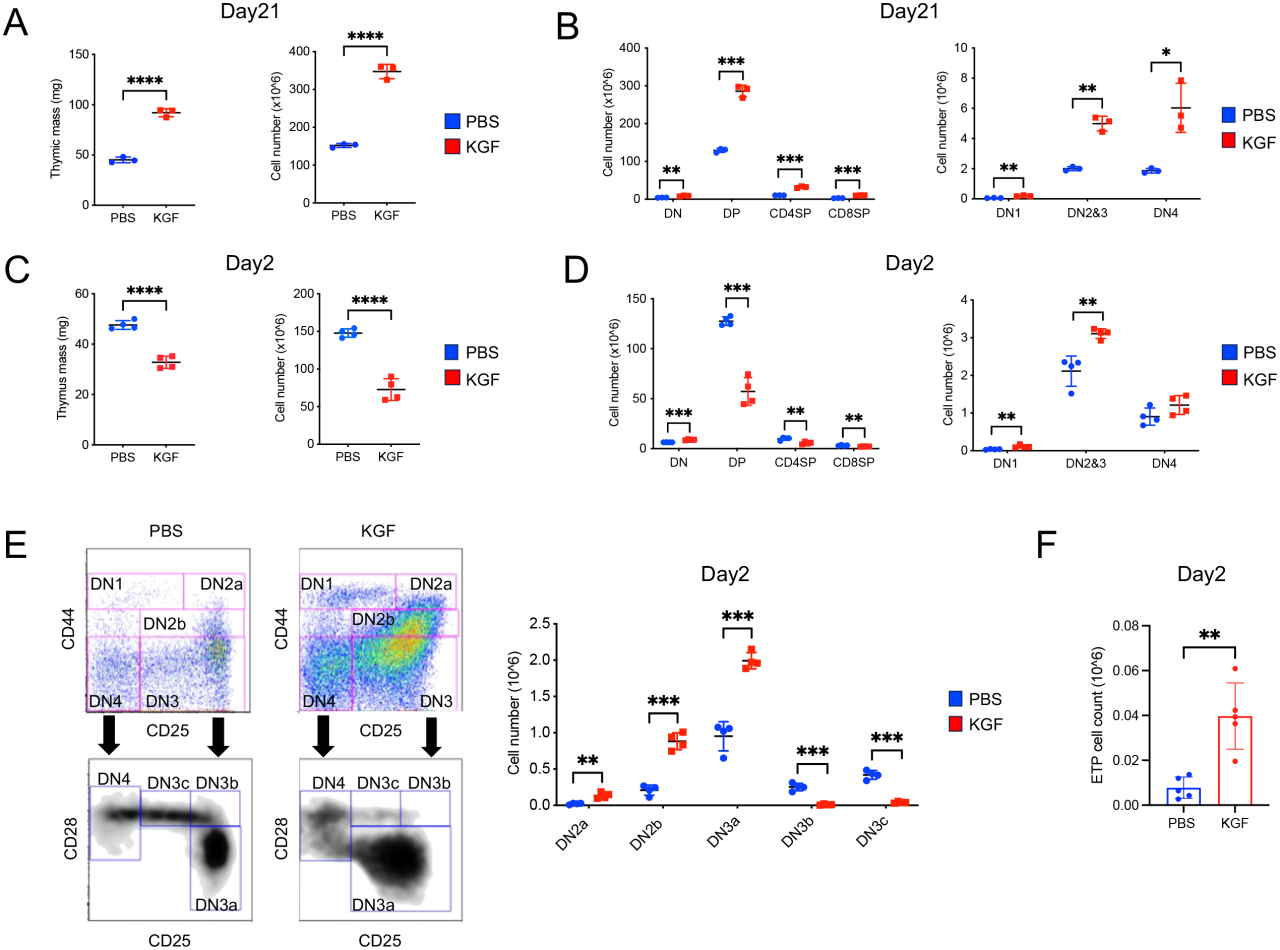
Exogenous KGF treatment alters thymocyte development and cellularity in adult thymus. **(A)** Thymus mass and thymocyte cellularity of PBS- and KGF-treated mice were assessed on day 21 following three consecutive daily doses (0.5mg/kg). **(B)** Absolute cell numbers of CD4^-^CD8^-^double negative (DN), CD4^+^CD8^+^double positive (DP), CD4^+^ single positive (CD4SP) and CD8^+^ single positive (CD8SP) thymocyte subsets (left), as well as DN1, DN2/3, and DN4 subsets (right) were quantified by flow cytometry in PBS- (blue) and KGF-treated (red) mice. **(C)** Thymus mass and thymocyte cellularity were evaluated on day 2 after three consecutive daily doses of KGF or PBS. **(D)** Absolute cell numbers of DN, DP, CD4SP, and CD8SP thymocyte subsets (left), along with DN1, DN2/3, and DN4 subsets (right), were assessed by flow cytometry on day 2 post-treatment. **(E)** Representative flow cytometry plots showing gateing strategies for identifying DN subpopulations and corresponding absolute thymocyte counts in PBS- (blue) and KGF-treated (red) mice at day 2 post-treatment. **(F)** Comparison of ETP cell number between PBS- and KGF-treated mice at day 2 post-treatment. Data in **(A, B)** are representative of three independent experiments; data in **(C–F)** are representative of more than three independent experiments. All data are presented as mean ± SD. Error bars indicate the standard deviation. Statistical significance was determined by unpaired two-tailed Student’s t-test: *p<0.05; **p<0.01; ***p<0.001; ****p<0.0001.

To investigate the effect of KGF on thymopoiesis and thymic epithelial cell (TEC) function, we compared thymus mass and thymocyte number in mice treated with KGF or PBS at various time points following three consecutive daily doses (0.5 mg/kg). As previously reported (29), we observed a dramatic increase in both thymus mass (~50%) and total thymocyte number (~100%) in KGF-treated mice compared to PBS-treated controls by day 21 (**Figure 1A**). This increase was attributed to an expansion across all thymocyte subsets (**Figure 1B**). However, an early analysis conducted on day 2 following the final KGF dose revealed a contrasting effect: both thymus mass and total thymocyte number were significantly reduced by approximately 32% and 50%, respectively, relative to controls (**Figure 1C**). This reduction was associated with a decline in post-β-selection thymocyte subsets, including DN3b (lineage-CD44^−^CD25^+^CD28^+^), DN3c (lineage-CD44^−^CD25^low^CD28^+^), double-positive (CD4^+^CD8^+^), CD4^+^ single-positive (CD8^−^CD4^+^), and CD8^+^ single-positive (CD8^+^CD4^−^) populations (**Figures 1D, E**). In contrast, KGF treatment resulted in a significant expansion of pre-β-selection thymocytes in DN1(lineage-CD44+CD25-), DN2a (lineage-CD44+CD25+c-kit^hi^), DN2b (lineage-CD44+CD25+c-kit^lo^) and DN3a (lineage-CD44-CD25+CD28-) subsets (**Figures 1D, E**). This expansion was accompanied by a marked increase in the earliest progenitor population, c-Kit^high^ ETP, which were nearly 2-fold higher in KGF-treated mice compared to controls (**Figure 1F**, **Supplementary Figure S1**). These opposing changes in pre- and post-β-selection thymocyte populations shortly after KGF treatment suggest that KGF may exert stage-specific effects on thymocyte differentiation, potentially influencing developmental progression at the β-selection checkpoint.

### 3.2 KGF blocks thymocyte development at the β-selection checkpoint

**Figure 2:**
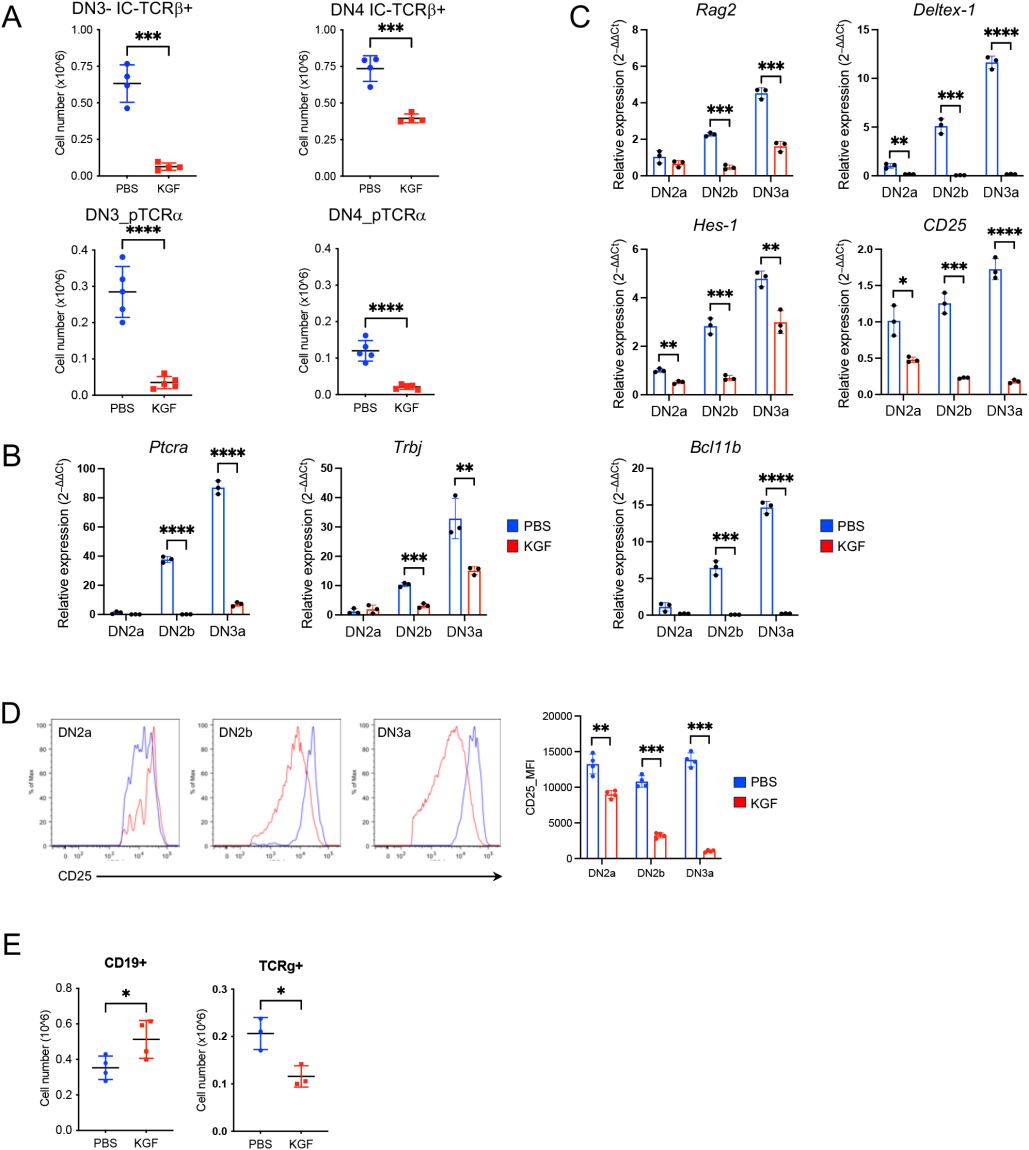
KGF inhibits pre-TCR expression and suppresses *Bcl11b* activation in DN thymocytes prior to β-selection. **(A)** Expression of TCRβ (n=4) and pre-TCRα (n=5) in DN3 and DN4 thymocyte subsets was assessed by flow cytometry on day 2 following PBS or KGF treatment. **(B, C)** mRNA levels of *Ptca* and *Trbj* genes **(B)**, as well as *Rag2*, *Deltex-1*, *Hes-1*, *CD25*, and *Bcl11b***(C)** were measured in sorted DN2a to DN3a subsets from PBS- (blue) and KGF-treated (red) mice using *TaqMan* gene expression assays. Data represent 3 independent experiments. **(D)** Cell surface CD25 expression on DN2a, DN2b, and DN3a subsets was quantified by flow cytometry using mean fluorescence intensity (MFI); representative histograms from four independent experiments are shown. **(E)** Numbers of B and TCRγ^+^ cells in PBS- (blue) and KGF-treated mice (red) (n=3-4) were quantified by flow cytometry on day 2 post-treatment. Data are presented as mean ± SD. Error bars indicate standard deviation. Statistical significance was determined using unpaired two-tailed Student’s t-test: *p<0.05; **p<0.01; ***p<0.001; ****p<0.0001.

Successful assembly of the pre-TCR complex is required for DN3a thymocytes to survive β-selection and continue differentiation into DN3b and later developmental stages. As such, we analyzed the expression of the pre-TCRα chain and TCRβ chain in DN thymocytes on day 2 following KGF treatment. The absolute number of TCRβ^+^ thymocytes within DN3 and DN4 subsets of KGF-treated mice decreased by approximately 90% and 50%, respectively, compared to their counterparts in PBS-treated controls (**Figure 2A**, **Supplementary Figure S2A**). Furthermore, the number of pre-TCRα^+^ thymocytes in the DN4 subset was reduced by more than 80% in KGF-treated mice, revealing a significant decrease in β-selection (**Figure 2A**, **Supplementary Figure S2B**). To further investigate these differences, we examined the transcription of *Ptcra* (pre-TCRα) and *Trbj* (T-cell receptor beta joining region). As expected, neither gene was detected in the DN2a subset in either control or KGF-treated mice (**Figure 2B**), In control mice, however, both genes showed a sharp upregulation starting at the DN2b stage and continuing through DN3a. In contrast, expression remained markedly low at these stages in KGF-treated mice (**Figure 2B**). Together, these findings support the notion that KGF blocks pre-TCR complex assembly by inhibiting transcriptional upregulation of key pre-TCR components, thereby impairing thymocyte progression through β-selection and reducing the number of post-β-selection thymocytes.

### 3.3 KGF disrupts Notch signaling and inhibits *Bcl11b* activation in early thymocytes

TCRβ rearrangement in thymocytes is initiated and mediated by recombinases, Rag1 and Rag2 (recombination activating gene 1 and 2) (30). Given the impaired TCRβ rearrangement observed in DN2b and DN3a thymocytes of KGF-treated mice, we directly examined *Rag2* expression. As expected, *Rag2* levels progressively increased from DN2a to DN3a in control mice (**Figure 2C**) (31). In contrast, KGF-treated mice showed no upregulation of *Rag2* until the DN3a stage, and expression levels remained approximately 60% lower than those in controls (**Figure 2C**), suggesting reduced Rag recombinase activity as a possible cause of impaired *Trb* rearrangement. The expression of *Ptcra* and *Rag* genes is known to be directly regulated by Notch1 signaling (32–35). Indeed, examination of direct Notch1 target genes revealed significantly lower expression of *Deltex-1*, and *Il2ra* (CD25) in DN2a–DN3a thymocytes of KGF-treated mice compared to PBS-treated controls (**Figure 2C**). Interestingly, *Hes1* was also reduced, though to a lesser extent, likely because its expression can be regulated by Hedgehog signaling independently of Notch (36). The reduction of Notch1-dependent genes was also evident at the protein level for CD25 (**Figure 2D**). Additionally, the *Bcl11b* gene, regulated by Notch1 in cooperation with transcription factors GATA3 and TCF-1 (7, 37, 38), was almost completely repressed in the same thymocyte subsets of KGF-treated mice (**Figure 2C**). While *Tcf7* was decreased in DN2a and DN2b subsets following KGF treatment, *Gata3* mRNA levels were comparable (**Supplementary Figure S3A**). Consistent with the diminished Notch1 signaling, which is known to divert early thymic progenitors (ETPs) toward the B cell lineage and block TCRγδ rearrangement (39), we observed a 1.5-fold increase in thymic B cell numbers and a 2-fold decrease in DN TCRγ^+^ thymocytes following KGF treatment (**Figure 2E**), but no significant changes were detected in Mac-1^+^/CD11b^+^, Gr-1, or NK1.1^+^ populations (**Supplementary Figure S3B**). Collectively, these findings indicate that KGF treatment disrupts Notch1 signaling, leading to impaired pre-TCR complex formation in DN2b and DN3a thymocytes.

### 3.4 KGF promotes uncommitted early thymocyte proliferation

**Figure 3:**
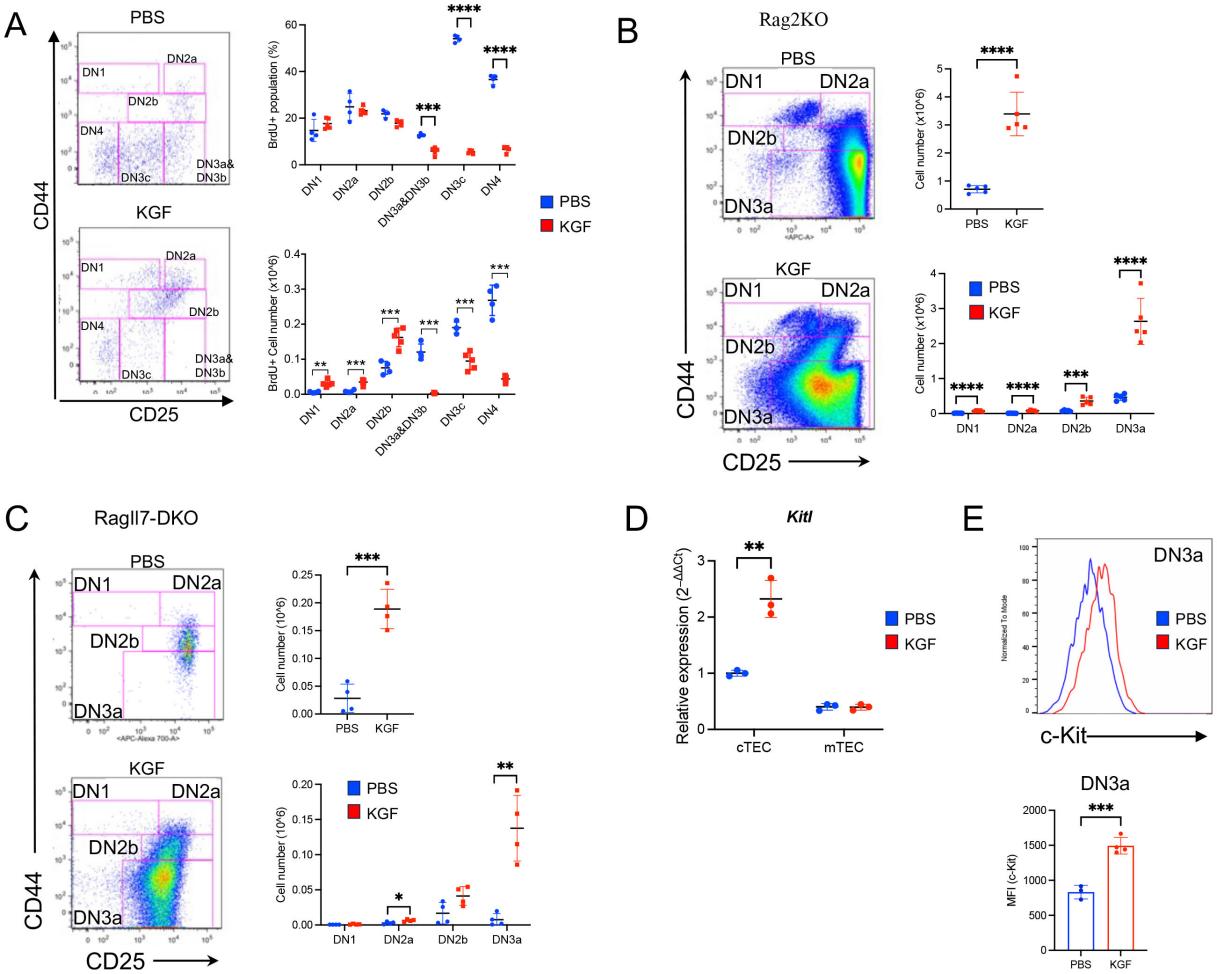
KGF treatment is associated with an expansion of pre-β-selection thymocytes. **(A)** The entry of DN thymocyte populations into S-phase of the cell cycle was assessed 2 hours after BrdU administration in PBS- (blue, n=4) and KGF-treated (red, n=5) mice (day 2 post-treatment). BrdU^+^ DN subsets were analyzed based on their CD44 and CD25 expression profiles (left). The frequencies (top right) and absolute numbers (bottom right) of BrdU^+^ thymocytes within each DN subset are shown. **(B)** DN thymocyte subsets in *Rag*2^−^/^−^ mice (Rag2KO) were analyzed based on CD44 and CD25 expression profiles on day 2 following PBS or KGF treatment (n=5) (left); and total thymocyte numbers and absolute counts of all DN subsets are presented (right). **(C)** DN subpopulations in *Rag*^−^/^−^*Il7*^−^/^−^ (RagIl7-DKO) mice (n=4) were analyzed on day 2 following three consecutive doses of PBS (blue) or KGF (red). Representative CD44/CD25 FACS plots of DN thymocytes (left), along with absolute numbers of total DN thymocytes (top right) and individual DN subsets (bottom right) are shown. **(D)***Kitl* mRNA levels were quantified in sorted cTECs and mTECs from PBS- (blue) and KGF- (red) treated wild-type mice. **(E)** Cell surface expression of c-Kit in DN3a thymocytes from PBS- (blue) and KGF-treated (red) mice was evaluated by flow cytometry. Representative histograms (top) from three independent experiments and MFI (bottom) are shown. Data represent the mean of three independent experiments and are presented as mean ± SD. Error bars represent standard deviation. Statistical significance was determined using unpaired two-tailed Student’s t-test: *p<0.05; **p<0.01; ***p<0.001; ****p<0.0001.

To assess the stage-specific impact of KGF, BrdU incorporation was measured in KGF- and PBS-treated mice at day 2 post-treatment (**Figure 3A**). While the frequency of BrdU^+^ cells amongst DN1, DN2a and DN2b thymocytes remained unchanged (**Figure 3A**, top right), the absolute number of BrdU^+^ cells in these subsets was 2- to 5-fold higher in KGF-treated mice compared to controls (**Figure 3A**, bottom right). In contrast, there was a marked KGF-induced reduction in cell cycle entry post–β-selection, with a complete KGF-mediated exit from the cell cycle by the DN3c stage, as shown by both absolute numbers and percentages (**Figure 3A**). Thus, preserved proliferation in early DN stages, coupled with a dramatic loss in later DN stages, likely accounts for the significant increase in DN2a-DN3a thymocyte numbers after KGF treatment (**Figures 1D, E**).

Because thymocyte growth prior to β-selection is regulated by TEC-derived IL-7 and Kit ligand (Kitl) (40–43), we next tested whether these signals mediate the effects of KGF. Il7 mRNA and intrathymic IL-7 levels were unchanged between KGF- and PBS-treated mice (**Supplementary Figures S4A, B**) but cell surface IL-7Rα (CD127) expression on DN2a-DN3a thymocytes was markedly decreased by KGF (**Supplementary Figures S4C, D**). Moreover, we assessed the impact of KGF in Rag2^−^/^−^ and Rag^−^/^−^Il7^−^/^−^ (DKO) mice, wherein thymocyte differentiation is blocked at the DN3a stage. In both Rag^−^/^−^ mice strains, KGF induced an expansion of DN thymocytes; with increases of 4.8- and 6.7-fold in Rag2^−^/^−^ and Rag^−^/^−^Il7^−^/^−^ mice, respectively (**Figures 3B, C**). These data indicate that early DN expansion after KGF treatment is independent of IL-7.

As regards c-Kit signaling, KGF increased Kitl expression in cTECs by ~2-fold (**Figure 3D**). Moreover, c-Kit levels were sustained at higher levels in DN3a thymocytes, whereas c-Kit was significantly downregulated in control DN3a thymocytes (**Figure 3E**). Together, these findings suggest that Kitl/c-Kit signaling supports the maintenance and expansion of early thymocytes in response to KGF.

### 3.5 KGF represses *Dll4* concomitantly with *Foxn1* attenuation in TECs

**Figure 4:**
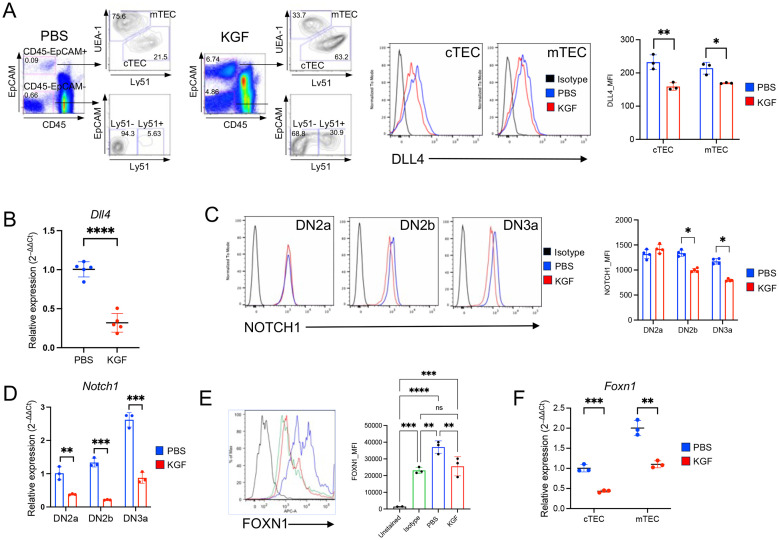
KGF reduces expression of DLL4 and its upstream transcriptional regulator FOXN1 in both cTECs and mTECs. **(A)** Thymic stromal subsets were defined based on EpCAM, Ly51, and UEA-1 expression profiles among CD45^-^ cells. Representative dot plots from PBS- and KGF-treated thymi (three independent experiments) are shown (left). EpCAM^+^ TECs were further subdivided into two major subsets: UEA-1^+^/Ly51^−^ medullary TECs (mTECs) and UEA-1^−^/Ly51^+^ cortical TECs (cTECs). DLL4 expression in cTECs and mTECs from PBS- (blue) and KGF-treated (red) wild type mice was evaluated by flow cytometry; representative histograms (middle) and mean fluorescence intensity (MFI) values (right) are shown. Isotype staining is shown in black. **(B)***Dll4* mRNA levels were measured in total TECs from PBS- (blue) and KGF-treated (red) mice. Data from three independent experiments are presented. **(C)** Cell surface expression of Notch1 receptor in DN2a-DN3a early thymocyte subsets from PBS- (blue) and KGF-treated (red) mice was evaluated by flow cytometry. Isotype staining is shown in black. Representative histograms from four independent experiments are shown (left), and MFI for each subset are presented (right). **(D)***Notch1* mRNA levels in DN2a-DN3a thymocyte subsets from PBS- (blue) and KGF-treated (red) mice are shown. **(E)** Intracellular FOXN1 expression in TECs from PBS- (blue) and KGF-treated (red) mice was assessed by flow cytometry. Representative histograms are shown, including an unstained control (black line) and IgG2b isotype control (green line) (left). Quantification of FOXN1 expression in total TECs by MFI is based on three independent experiments. **(F)***Foxn1* mRNA levels in sorted TEC subsets from PBS- (blue) and KGF- treated (red) mice were also evaluated and quantitative results are shown. Data are presented as mean ± SD. Error bars indicate standard deviation. Statistical significance between two groups was determined using unpaired two-tailed t-tests. One-way ANOVA was applied for comparison of 3 and more groups and p-values were determined by Tukey’s multiple comparisons test: *p<0.05; **p<0.01; ***p<0.001; ****p<0.0001; ns = not significant.

Consistent with previous studies, our investigation of KGFR/FGFR2IIIb expression at both the protein and transcript levels confirmed its exclusive localization in TECs (**Supplementary Figure S5**) (10, 11, 24). Given that KGF treatment disrupted Notch signaling in DN2a–DN3a thymocytes, we next examined the expression of *Dll4*, the primary Notch ligand, in TECs. *Dll4* is primarily expressed in cTEC, as compared to mTEC, but its expression in the former decreases with age (44, 45). Indeed, DLL4 expression was significantly reduced in both cTECs and mTECs of KGF-treated mice compared to PBS-treated controls (**Figure 4A**). This reduction was also observed in the total TEC population at the transcript level (**Figure 4B**). The reduced DLL4 expression in TECs correlated with lower Notch1 expression in DN2a–DN3a thymocytes (**Figures 4C, D**), consistent with previous studies showing that abrogation of Notch signaling in thymocytes leads to a downregulation of Notch1 receptor expression (46, 47). Because FOXN1 is a critical transcription factor for TEC function and is required for *Dll4* expression (16, 48), we next assessed the impact of KGF on *Foxn1* expression. Notably, KGF treatment significantly decreased *Foxn1* expression in both cTECs and mTECs at the protein and transcript levels (**Figures 4E, F**). Together, these data strongly suggest that KGF impairs Notch signaling in early thymocytes by acting on TECs to downregulate *Foxn1*, thereby attenuating *Dll4* expression.

### 3.6 KGF suppression of *Wnt* ligand expression is associated with decreased Wnt/β-catenin signaling

**Figure 5:**
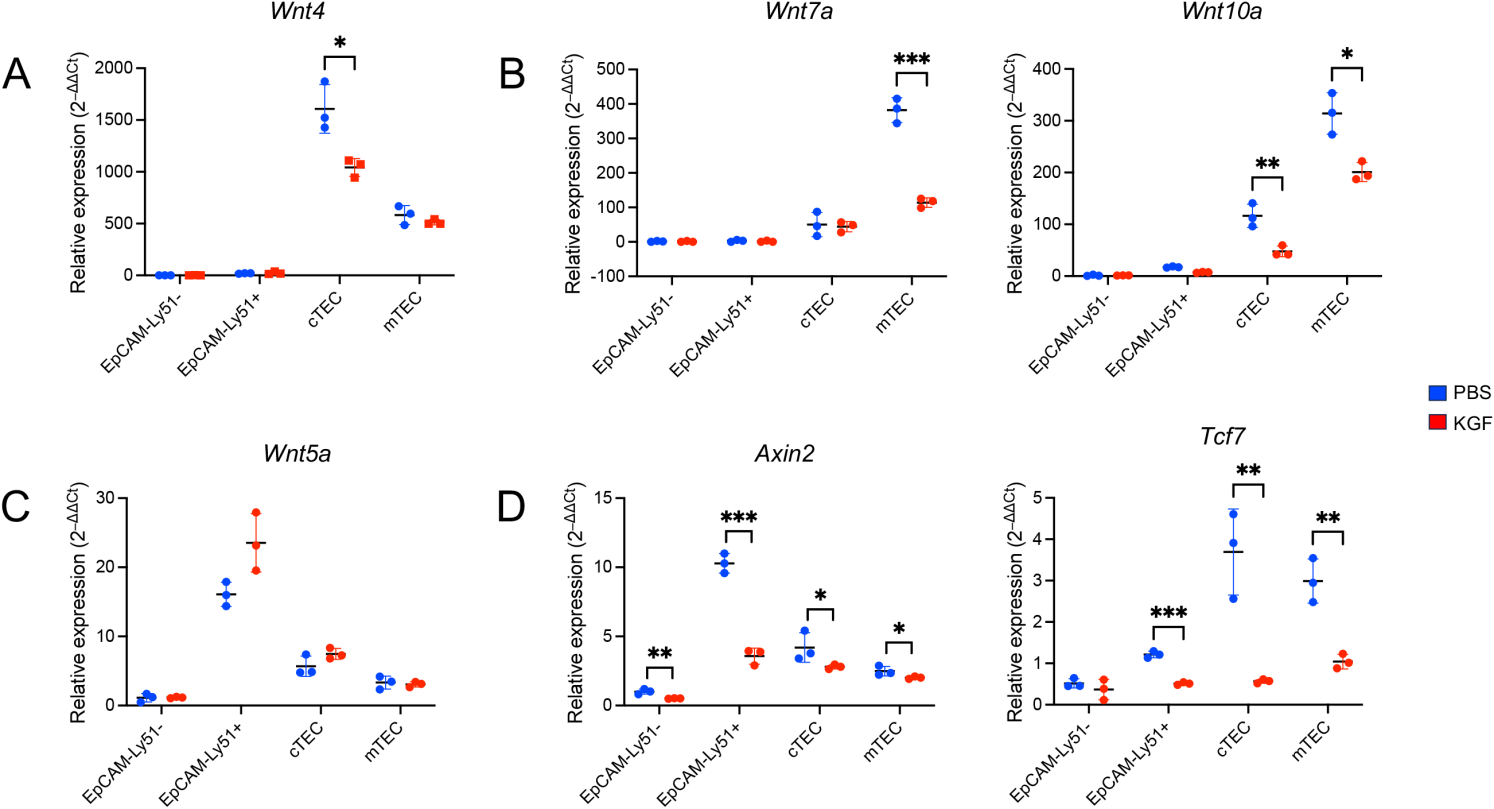
KGF suppresses Wnt/β-catenin signaling and reduces Wnt ligand expression in TECs. Expression of Wnt ligands *Wnt4***(A)**, *Wnt7a and Wnt10a***(B)**, *Wnt5a***(C)**, and Wnt/β-catenin signaling target genes *Axin2* and *Tcf7***(D)** were evaluated by *Taqman* gene expression assays in sorted thymic stromal subsets from PBS- (blue, n=3) and KGF-treated (red, n=3) mice; EpCAM^-^Ly51^-^ (Endothelial), EpCAM^-^Ly51^+^ (Mesenchymal), cTEC (EpCAM^+^UEA-1^−^/Ly51^+^) and mTEC (EpCAM^+^UEA-1^+^/Ly51^−^). Data are presented as mean ± SD. Error bars represent the standard deviation from three independent experiments. Statistical significance was determined using an unpaired two-tailed Student’s t-test: *p<0.05; **p<0.01; ***p<0.001.

β-catenin-dependent canonical Wnt signaling plays a critical role in the regulation of *Foxn1* expression in both embryonic and adult thymus (17–20). To explore whether KGF-mediated downregulation of *Foxn1* in TECs involves the Wnt pathway, we examined the impact of KGF on the expression of three key TEC-specific Wnt ligands: Wnt4, Wnt7a, and Wnt10a. As previously reported (17, 21), we first confirmed the TEC-specific expression of these ligands. Indeed, we found Wnt4 mRNA levels were approximately threefold higher in cTECs than in mTECs, whereas Wnt7a and Wnt10a were more highly expressed in mTECs (**Figures 5A, B**).

Following KGF treatment, we observed a 30% reduction in *Wnt4* transcripts in cTECs, with no significant change in mTECs (**Figure 5A**). Wnt7a levels were reduced by 70% in mTECs, but remained unchanged in cTECs. Wnt10a expression was decreased in both TEC subsets, with a 60% decrease observed in cTECs and a 40% decrease in mTECs (**Figure 5B**). These findings indicate that KGF treatment leads to a subtype-specific downregulation of all three major Wnt ligands in TECs. In contrast, the expression of Wnt5a, a ligand restricted to EpCAM^−^Ly51^+^ stromal cells, remained unchanged after KGF treatment (**Figure 5C**), consistent with the restricted expression of KGFR to TECs (**Supplementary Figure S5**).

To determine whether this reduction in Wnt ligand expression corresponded with an alteration of Wnt/β-catenin signaling, we assessed the expression of two canonical Wnt/β-catenin signaling target genes, *Axin2* and *Tcf7* (49, 50). Notably, both genes were significantly downregulated in both TEC subsets as well as in EpCAM^−^ mesenchymal cells following KGF treatment (**Figure 5D**), suggesting that KGF-mediated suppression of Wnt ligand expression leads to attenuated Wnt/β-catenin signaling within TECs. The cross-talk between TECs and mesenchymal cells plays a critical role during thymic organogenesis and in maintaining thymic function and architecture (2, 51). The observed downregulation of *Axin2* and *Tcf7* in KGFR-negative EpCAM^−^ mesenchymal cells following KGF treatment likely reflects this intercellular interaction within the thymic microenvironment.

### 3.7 KGF downregulates *Foxn1* expression in TECs through suppression of Wnt/β-catenin signaling

**Figure 6:**
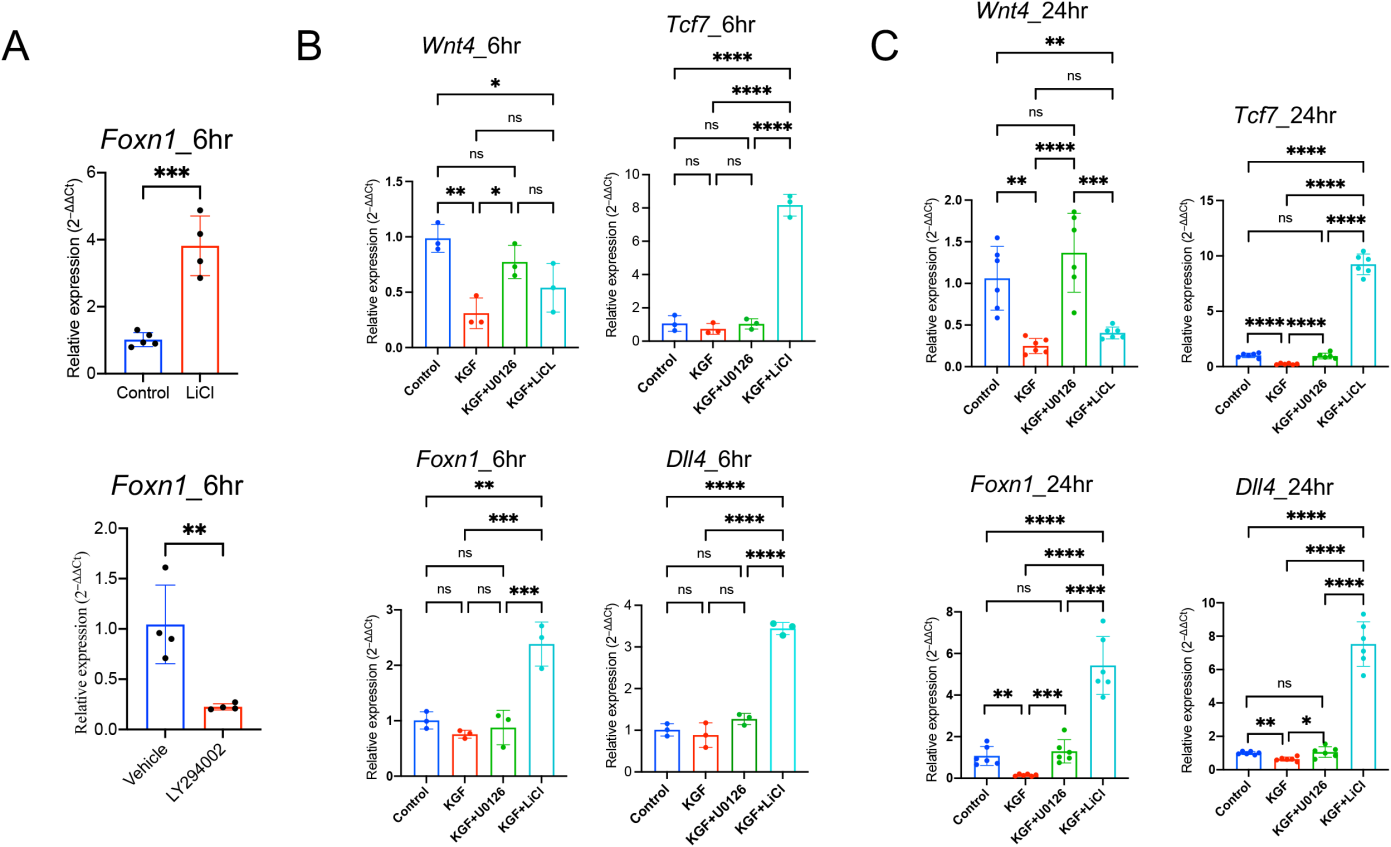
KGF regulates *Foxn1* expression in TECs through Wnt/β-catenin signaling. **(A)** Effect of LiCl and LY294002 on *Foxn1* transcription in TE-71 cells. Data represent four experimental replicates. **(B)** Expression of the Wnt ligands *Wnt4*, *Foxn1, Dll4 and* Wnt/β-catenin signaling target gene *Tcf7*, was assessed in TE-71*^kgfr^* cells cultured for 6 hours with vehicle control (blue), KGF (red), KGF+U0126 (green), or KGF+LiCl (cyan). Data represent three experimental replicates. **(C)** Gene expression was evaluated under the same conditions as in **(B)** after 24 hours of culture. Data represent six experimental replicates. All data are presented as mean ± SD. Error bars indicate the standard deviation. Statistical significance for **(A)** was determined using an unpaired two-tailed Student’s t-test: **p<0.01; ***p<0.001. For **(B**, **C)**, one-way ANOVA followed by Tukey’s multiple comparisons test was used: *p<0.05; **p<0.01; ***p<0.001; ****p<0.0001; ns = not significant.

Our observation that KGF suppresses Wnt/β-catenin signaling and downregulates *Foxn1* expression in TECs prompted us to investigate whether the regulation of *Foxn1* expression by KGF is directly mediated through the Wnt/β-catenin signaling pathway. To assess whether *Foxn1* expression is responsive to changes in this signaling, we utilized TE-71 cells. While this is an mTEC line (52), it retains key cTEC features, including expression of *Epcam*, *Foxn1, Kgfr*(*Fgfr2IIIb*), *Wnt4, Krt5* and *Krt8*, with absent expression of the fibroblast marker *Fgfr2IIIc* (**Supplementary Figures S6A, B**). Treatment with lithium chloride (LiCl), a GSK-3β inhibitor that enhances Wnt/β-catenin signaling by preventing β-catenin phosphorylation and degradation (53), led to a threefold increase in *Foxn1* mRNA levels within six hours (**Figure 6A**). Conversely, inhibition of the pathway using LY294002, a PI3K inhibitor that indirectly enhances GSK-3β activity (54), resulted in a 70% reduction in *Foxn1* transcript levels (**Figure 6A**). These results indicate that *Foxn1* expression is positively regulated by Wnt/β-catenin signaling in TECs.

To further investigate the direct role of KGF, we established TE-71*^kgfr^* cells with elevated KGFR expression through transgenic overexpression of *Kgfr* (**Supplementary Figure S7**). KGF treatment of TE-71*^kgfr^* cells led to a significant reduction in *Wnt4* mRNA, as early as six hours (**Figure 6B**). By 24h, *Foxn1* and *Dll4* transcripts were reduced by 40–80%, accompanied by decreased expression of the Wnt/β-catenin target gene *Tcf7* (**Figure 6C**). These findings suggest that early suppression of Wnt4 may trigger the subsequent KGF-mediated inhibition of Wnt/β-catenin signaling.

Furthermore, LiCl treatment reversed the inhibitory effects of KGF and enhanced Wnt/β-catenin signaling, leading to increased expression of *Tcf7, Foxn1* and *Dll4* in TE-71*^kgfr^* cells (**Figures 6B, C**), despite the persistent suppression of *Wnt4* transcription (**Figures 6B, C**). Together, these results support the hypothesis that KGF inhibits *Foxn1* expression and Wnt/β-catenin signaling primarily through the suppression of TEC-derived Wnt ligands.

### 3.8 KGF-induced TE-71 proliferation is dependent on the MAPK/ERK signaling

**Figure 7:**
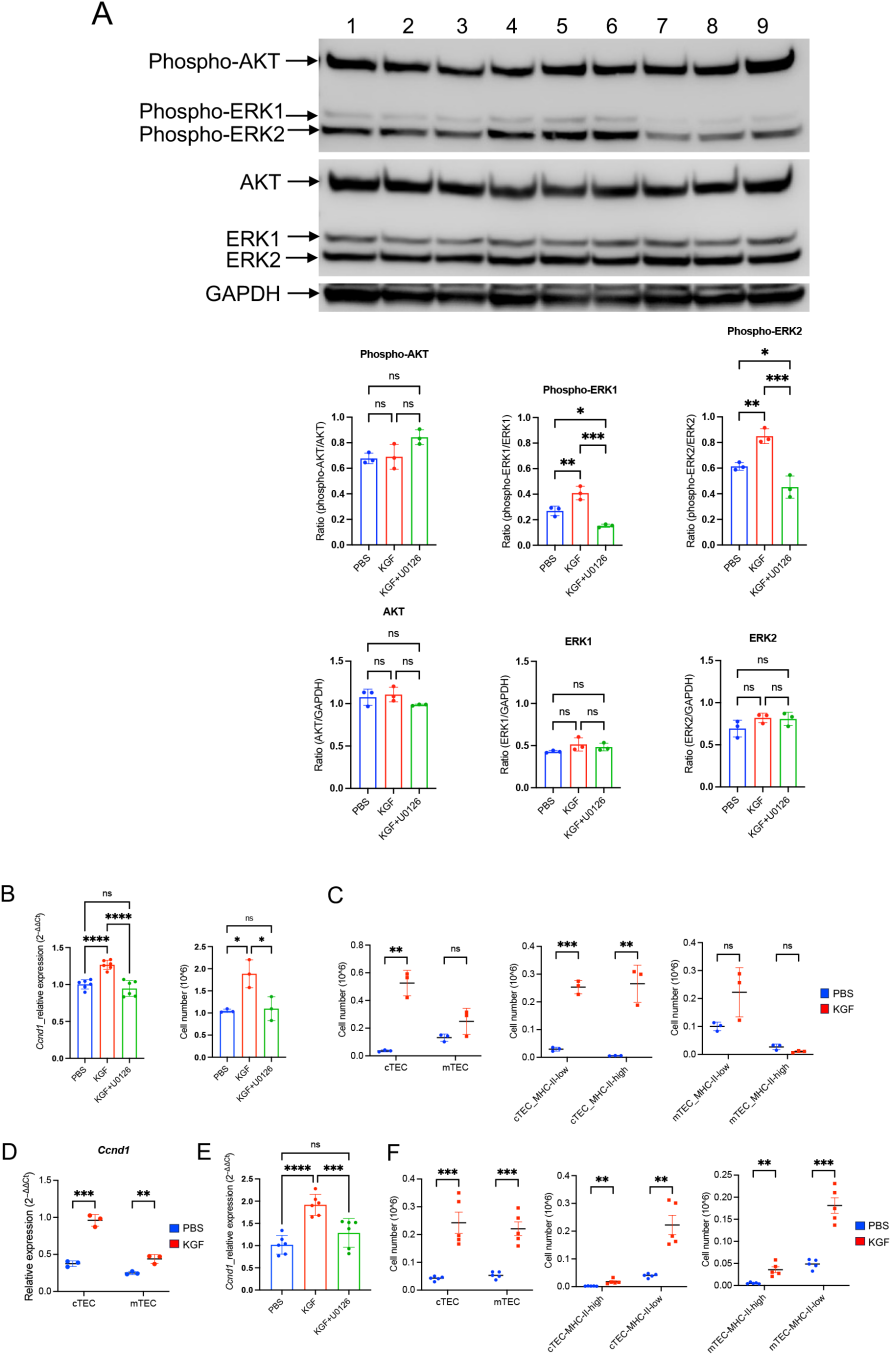
KGF promotes proliferation of TE-71 cells and primary TECs through activation of the MAPK/ERK signaling pathway. **(A)** Phosphorylation of AKT (Ser473) and ERK1/2 (Thr202/Tyr204) was evaluated in TE-71*^kgfr^* cells cultured for 6 hours with vehicle (blue), KGF (red), and KGF+U0126 (green). Representative immunoblots from three independent experiments are shown: lanes 1-3 (vehicle), lanes 4-6 (KGF), and lanes 7-9 (KGF+U0126) (top). Quantification of band intensities is presented (bottom). **(B)** Cyclin D1/*Ccnd1* mRNA levels (left) and total cell numbers (right) were analyzed in TE-71*^kgfr^* cells cultured with vehicle (blue), KGF (red), or KGF+U0126 (green) for 72 hours. **(C)** Absolute numbers of cTECs and mTECs (left), MHC-II^high^ and MHC-II^low^ cTECs (middle), and MHC-II^high^ and MHC-II^low^ mTECs (right) were compared between PBS-treated (blue, n=3) and KGF-treated (red, n=3) wild-type mice on day 2 following three consecutive daily doses. **(D)***Ccnd1* expression in sorted cTEC and mTEC populations from PBS- (blue, n=3) and KGF-treated (red, n=3) mice [as in **(C)**] is shown. **(E)***Ccnd1* expression levels were evaluated in thymic fragments cultured with vehicle, KGF, and KGF+U0126 for 24 hours. Data represent three independent experiments. **(F)** TEC numbers in PBS-treated (blue, n=5) and KGF-treated (red, n=5) mice were assessed 6 weeks after three consecutive doses of treatment. Cell numbers of cTEC and mTEC (left), MHC-II^high^ and MHC-II^low^ cTEC (middle), MHC-II^high^ and MHC-II^low^ mTEC (right) are shown. Data are presented as mean ± SD. Error bars indicate standard deviation. Statistical significance for comparisons between two groups was determined using an unpaired two-tailed Student’s t-test (**p<0.01; ***p<0.001). For comparisons among three different conditions, one-way ANOVA followed by Tukey’s multiple comparisons test was used: *p<0.05; ***p<0.001; ****p<0.0001; ns, not significant. Full-length, uncropped blots corresponding to those in panel **(A)** are provided in **Supplementary Figures S8**, **S9**.

As KGF binding to KGFR is known to activate MAPK/ERK or PI3/AKT signaling in a cell-type dependent manner (55), we examined the activity of both pathways in TE-71*^kgfr^* cells upon KGF stimulation. Within 6 hours of KGF treatment, a marked increase in ERK1/2 phosphorylation (Thr202/Tyr204) was observed compared to control conditions (PBS), whereas no change in AKT phosphorylation (Ser473) was detected (**Figure 7A**). These findings indicate that KGF/KGFR signaling specifically activates the downstream MAPK/ERK pathway in TE-71 cells. Given that *Ccnd1* (cyclin D1), a known downstream target of MAPK/ERK signaling, plays a key role in regulating proliferation across various cell types (56–58), we used its expression as a readout of ERK pathway activation in TE-71*^kgfr^* cells. After 3 days of KGF treatment, *Ccnd1* mRNA levels increased by 1.3-fold relative to vehicle controls, and this upregulation was completely abolished by addition of the ERK inhibitor U0126 to the culture (**Figure 7B**). Correspondingly, cell numbers increased by 1.8-fold following KGF stimulation, an effect also dependent on ERK activity (**Figure 7B**). These results support the conclusion that the proliferative effect of KGF on TE-71^kgfr^ cells is mediated specifically through MAPK/ERK signaling.

To evaluate the *in-vivo* impact of KGF, we examined TEC expansion in KGF-treated mice. Analysis of thymic stromal subpopulations revealed a striking 15-fold increase in the absolute number of cTECs by day 2 post-treatment compared to PBS-treated control mice (**Figure 7C**). This expansion was evident in both immature MHC-II^low^ (major histocompatibility complex class II) progenitors and MHC-II^high^ mature cTECs (**Figure 7C**). In contrast, no significant KGF-induced proliferative effect was observed in mTECs, regardless of their MHC-II status, although a modest increase in MHC-II^low^ mTEC progenitors was noted in KGF-treated mice (**Figure 7C**). Consistent with the observed expansion, *Ccnd1* mRNA levels increased 3-fold in cTECs and 1.76-fold in mTECs (**Figure 7D**). Moreover, *Ccnd1* expression was elevated 2-fold in whole thymic fragments 24 hours following KGF treatment, and this increase was abrogated by the ERK pathway inhibitor U0126 (**Figure 7E**), implicating the KGF/KGFR–MAPK/ERK signaling axis in mediating these effects. Importantly, the KGF-induced expansion of the thymic stromal compartment with a 4- to 5-fold increase in both cTECs and mTECs, was observed at six weeks after KGF treatment (**Figure 7F**). These findings underscore the central role of KGF-mediated MAPK/ERK signaling in regulating TEC proliferation and long-term thymic stromal remodeling.

### 3.9 KGF-induced downregulation of Wnt/β-catenin signaling is dependent on recruitment of the MAPK/ERK signaling pathway

**Figure 8:**
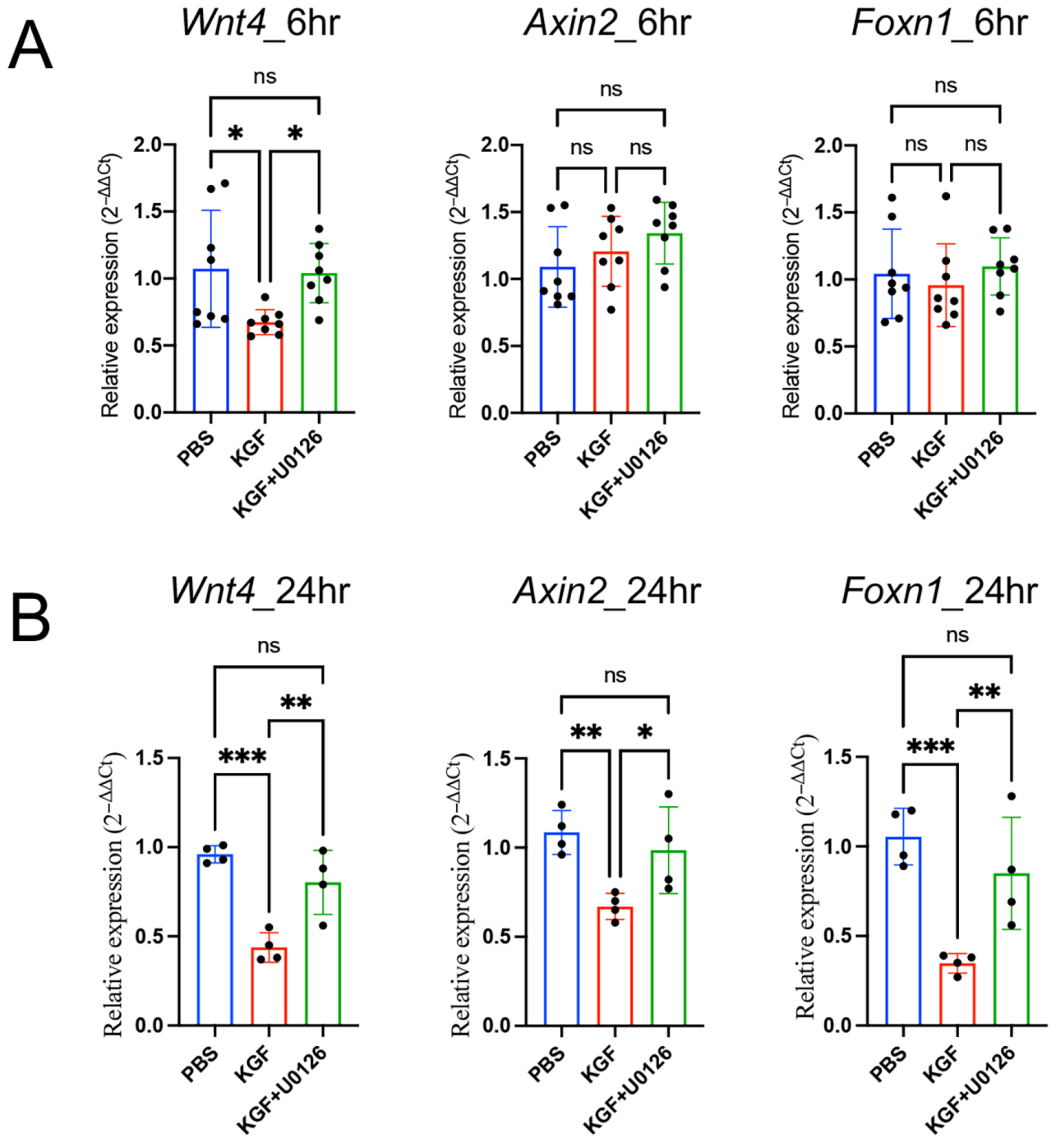
KGF regulates *Foxn1* expression in thymic fragments through Wnt/β-catenin signaling. **(A, B)** Expression of the Wnt ligand *Wnt4* and Wnt/β-catenin target genes *Axin2* and *Foxn1* was assessed in thymic fragments cultured with vehicle (blue), KGF (red), and KGF+U0126 (green) for 6 hours **(A)** and 24 hours **(B)**. Data are presented as mean ± SD. Error bars represent standard deviation. Statistical significance for comparisons among the three culture conditions was determined by one-way ANOVA followed by Tukey’s multiple comparisons test: *p<0.05; **p<0.01; ***p<0.001. ns, not significant.

To determine whether KGF regulates Wnt/β-catenin signaling through the MAPK/ERK pathway, we used U0126 to block the MAPK/ERK pathway in KGF-treated TE-71*^kgfr^* cells. U0126 inhibited the KGF-mediated downregulation of *Wnt4* transcription at 6 hours and prevented the subsequent repression of *Foxn1*, *Dll4* and *Tcf7* expressions by 24 hours (**Figures 6B, C**). Similarly, in thymic fragments, U0126 also inhibited the KGF-induced repression of *Wnt4* expression by 6 hours (**Figure 8A**). Furthermore, in the presence of U0126, KGF no longer reduced the expression of downstream Wnt/β-catenin targets, *Foxn1* and *Axin*2 (**Figure 8B**). Taken together, these findings highlight that KGF modulates Wnt/β-catenin signaling via the MAPK/ERK pathway, thereby influencing thymocyte proliferation and differentiation by regulating both TEC function and proliferation.

## 4 Discussion

Within the thymus, KGFR and its ligands FGF10 and FGF7 (also known as KGF) are critical for TEC development. Genetic ablation of *Kgfr* or *Fgf10* results in severe thymic hypoplasia due to impaired epithelial cell expansion, underscoring the essential role of KGFR-dependent signaling in thymic organogenesis (22, 23). Conversely, administration of exogenous KGF has been shown to promote TEC proliferation and drive thymocyte expansion as early as 1 week following treatment (25, 59). In addition, KGF confers protection to the thymus under conditions of stress, including irradiation, chemotherapy, and graft-versus-host disease, with these effects largely attributed to its activity in KGFR-expressing TECs (29, 59). Mechanistically, KGF has been proposed to enhance thymopoiesis by upregulating *Bmp2* and *Bmp4* expression and by promoting the proliferation of IL-7-producing TECs (25, 60). However, the intracellular signaling cascades activated downstream of KGFR and their specific roles in modulating thymopoiesis have remained poorly defined.

In this study, we focused on the early molecular consequences of KGF treatment, revealing a previously unrecognized transient suppression of the evolutionary conserved Wnt/β-catenin–*Foxn1*–*Dll4* axis in TECs. A rapid KGF-induced activation of the MAPK/ERK pathway led to proliferation of TECs via cyclin D1/*Ccnd1* induction, but concurrently suppressed expression of TEC-derived Wnt ligands, particularly Wnt4, resulting in inhibition of canonical Wnt signaling. This, in turn, downregulated the TEC master transcription factor FOXN1 and its downstream target DLL4, a key Notch ligand required for thymocyte progression.

Downregulation of DLL4 expression disrupted Notch signaling in thymocytes, suppressing expression of key downstream targets— *Bcl11b*, *Ptcra*, and *Rag2*—in pre-β-selection thymocytes. This resulted in suppression of pre-TCRα expression and hindered TCRβ chain rearrangement, thereby blocking thymocyte progression through the β-selection checkpoint. Together, these findings delineate a signaling cascade—KGF → MAPK/ERK → *Wnt* suppression → *Foxn1* downregulation → *Dll4* reduction → Notch signaling loss— that transiently disrupts thymopoiesis in the context of TEC proliferation. Based on our data, we hypothesize that intracellular Notch1 (ICN1) induction is impaired by KGF treatment, which remains an important question for future investigation. Notably, in parallel with this disruption in thymopoiesis and TEC proliferation, we observed increased proliferation of early thymocyte subsets, including ETPs, DN2a, and DN2b, alongside elevated expression of *Kitl* in TECs. This suggests that KGF may also regulate *Kitl* expression and exert multifaceted effects on thymic function.

Importantly, these early effects were biphasic and transient. By three weeks post-treatment, thymic mass and thymocyte cellularity had more than doubled, and expansion of cTECs and mTECs persisted for at least six weeks after cessation of KGF. Thus, KGF initiates a short-lived suppression of *Foxn1* and *Dll4*, followed by a durable expansion of the TEC compartment that ultimately supports increased thymopoiesis. This biphasic effect reconciles the paradoxical early decline and later rebound in thymocyte production observed *in vivo*.

KGF binding to its receptor KGFR primarily activates either the PI3K/AKT or MAPK/ERK signaling pathways, depending on the pre-existing intracellular environment of epithelial cells (55). In our study, we identified a specific KGF-mediated activation of the MAPK/ERK pathway in TECs. This was demonstrated in both TE-71*^kgfr^* and thymic fragment cultures, where KGF-induced downregulation of *Wnt4* gene expression at 6 hours was completely blocked by the ERK phosphorylation inhibitor U0126. Furthermore, under ERK-inhibited conditions, KGF failed to induce *Ccnd*1 expression or promote proliferation of TE-71 cells. These findings indicate that KGF exerts its mitogenic effects on TECs primarily through the KGFR–MAPK/ERK signaling axis.

FOXN1 plays a pivotal role in the molecular machinery that governs TEC differentiation and function, in part by regulating the expression of key target genes such as *Dll4* (15, 16, 48). Our findings suggest that KGF regulates *Foxn1* by suppressing Wnt ligand expression. Canonical Wnt/β-catenin signaling is a well established regulator of thymic organogenesis (17–19), but its contribution in the adult thymus remains less clear. Analyses of Wnt ligands in TEC subsets reveals distinct expression patterns, with Wnt4 expressed at higher level in cTECs and Wnt10a in mTECs (44). KGF treatment markedly reduces expression of both ligands, leading to diminished expression of the Wnt/β-catenin target genes *Axin2* and *Tcf7* and subsequent downregulation of *Foxn1* and *Dll4* across TEC subsets. Consistent with the subset-specific pattern of Wnt ligand expression, KGF induced an earlier proliferative response in cTECs, whereas mTEC proliferation was delayed, further underscoring the distinct kinetics with which these two TEC subsets respond to KGF. The approximately four-fold higher expression of *Kgfr* in cTECs compared with mTECs (**Supplementary Figure S5C**) likely contributes to the more robust proliferation observed in cTECs.

WNT4 is known to activate both canonical (β-catenin–dependent) and non-canonical Wnt pathways (61–63). While our data indicate that β-catenin–dependent Wnt signaling is a driver of *Foxn1* expression in the adult TECs, given the dual signaling potential of Wnt4 (62, 63), contributions from non-canonical pathways cannot be excluded. The precise interplay between canonical and non-canonical Wnt signaling in adult TECs—and how this balance is modulated by KGF—remains to be elucidated.

Collectively, our findings suggest that the KGF-induced arrest of early T-cell development at the β-selection checkpoint is primarily mediated by cTECs. We find that KGF downregulates *Foxn1*/FOXN1 expression and attenuates Wnt/β-catenin signaling in mTECs but it remains to be determined whether functional alterations due to these changes directly contribute to the disruption of early thymocyte development. In particular, whether KGF alters mTEC production of key chemokines such as CCL19 or CCL21, which are critical for guiding thymocyte migration toward the medulla, and whether such changes indirectly disrupt early thymocyte development warrants further investigation.

Our study also helps contextualize the discrepant outcomes of KGF treatment in preclinical models versus human clinical trials. While animal studies consistently reported thymic recovery and enhanced T cell output following KGF administration (25, 29, 59, 60), recent clinical trials have failed to demonstrate improved thymopoiesis in patients with multiple sclerosis (26) and HIV (27). In one trial, KGF treatment following lymphodepletion paradoxically reduced thymic output (26). Our findings suggest that the timing and cellular context of KGF signaling, particularly its early suppressive effect on TEC-mediated Notch signaling, may underlie these mixed outcomes. KGF drives robust proliferation and significant expansion of early ETPs through DN3a thymocytes and promotes proliferation of both cTECs and mTECs (upregulation of Ccnd1 expression). These early events likely set the stage for the well-documented effects of KGF observed at later time points post-treatment, suggesting that transient modulation of TEC signaling can ultimately enhance thymic function. These insights highlight the importance of temporal regulation and signaling context in designing KGF-based therapies. They also underscore that both stimulation of early thymocyte proliferation and induction of TEC proliferation may be essential to achieve optimal immune reconstitution.

## Data Availability

The datasets presented in this study can be found in online repositories. The names of the repository/repositories and accession number(s) can be found in the article/**Supplementary Material**.
