# Supplementary material for: Disruption of Notch signaling by KGF induces a developmental pause in thymocytes
Source: Front Immunol. 2025 Nov 21;16:1675823. doi: 10.3389/fimmu.2025.1675823 (PMC12678383; doi:10.3389/fimmu.2025.1675823)
Supplement: Supplementary file 1 [file Presentation1.pdf]

Online Supplemental Material

Figure S1

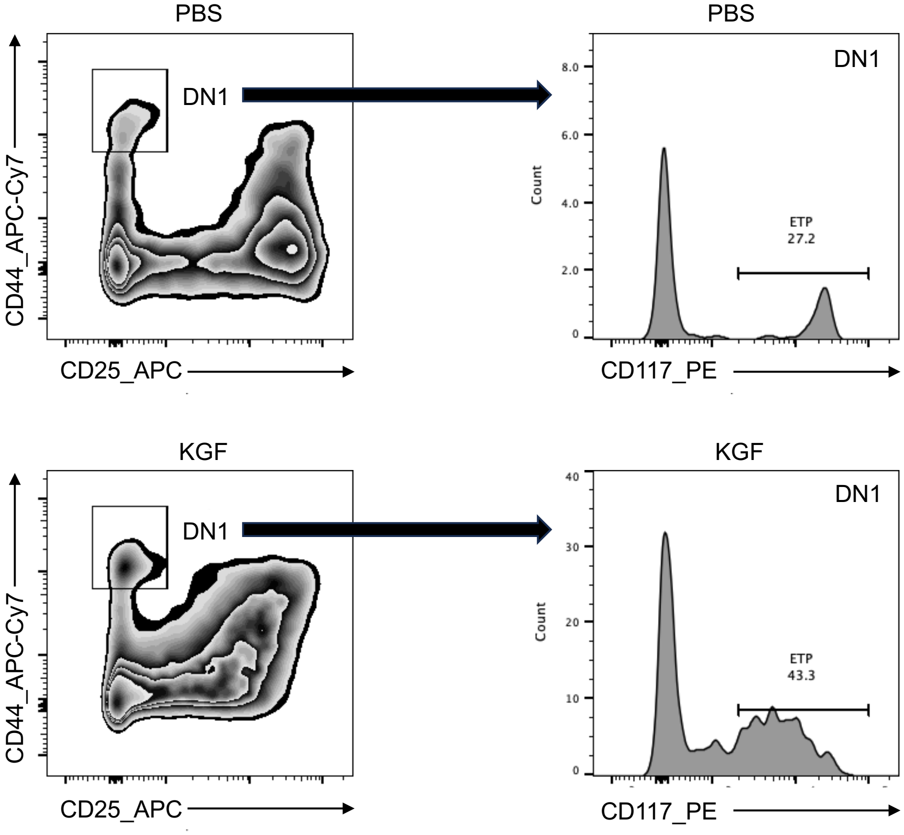

Flow cytometry gates for analysis of ETPs. Representative flow cytometry plots of DN thymocytes from PBS- and KGF-treated mice, 2 days following treatment. DN1 thymocytes were gated as $Lin^{-}CD25^{-}CD44^{+}$, and ETPs were identified as $cKit^{+}$ (CD117) cells within the DN1 subset.

Figure S2

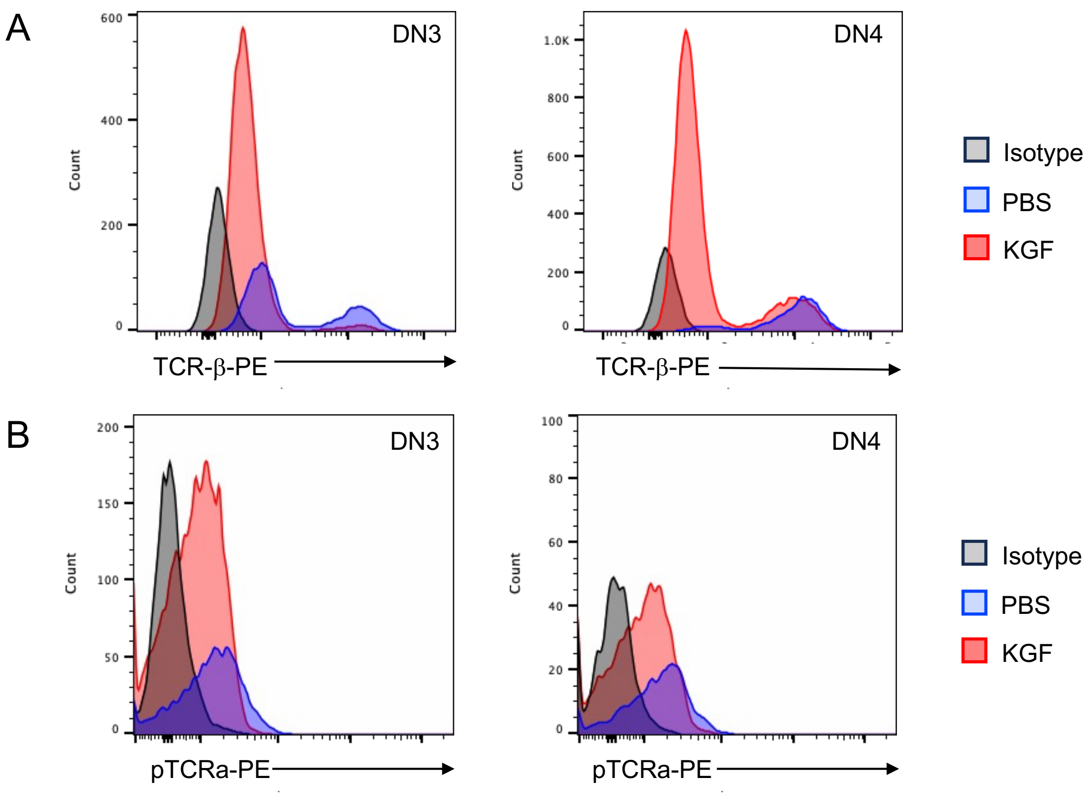

Flow cytometry analysis of TCR-β and pre-TCRα expression in DN3 and DN4 thymocytes. Representative flow cytometry plots showing the expression of TCR-β (A) and pre-TCRα (B) in DN3 (left) and DN4 (right) thymocyte subsets from C57BL/6 mice treated with PBS (blue line) or KGF (red line) on day 2 following three consecutive daily doses. Black histograms represent isotype control staining.

Figure S3

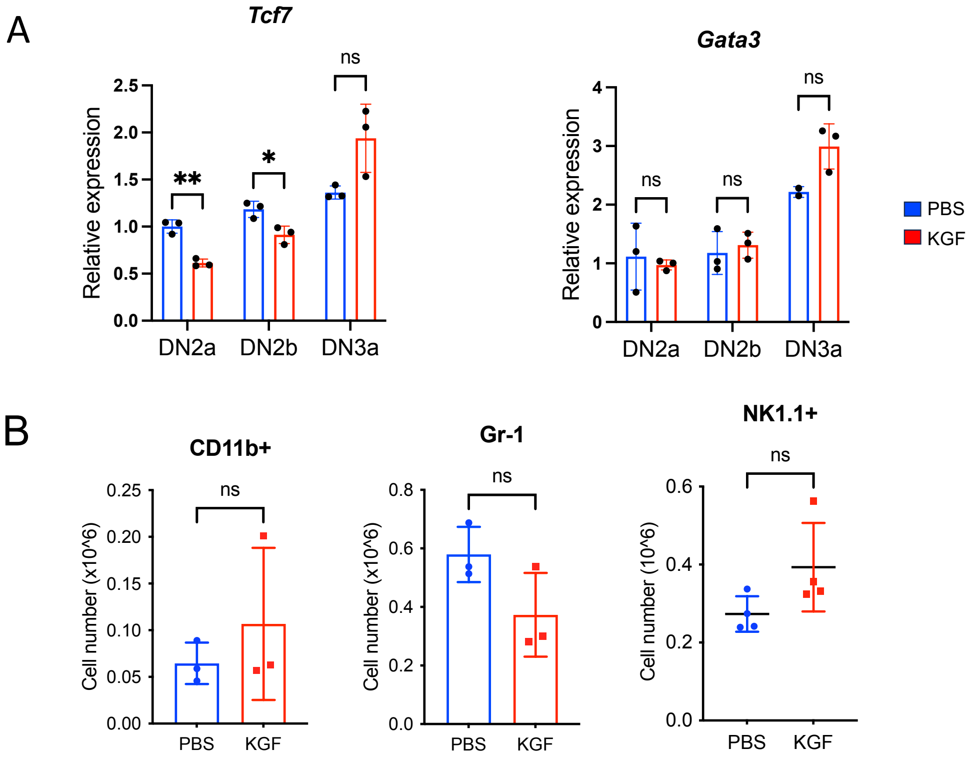

Characterization of non–T-cell populations and Tcf7/Gata3 expression in thymus. (A) Tcf7 and Gata3 mRNA levels in sorted DN2a to DN3a subsets were measured by TaqMan gene expression assays. (B) Total numbers of CD11b+, Gr-1+ and NK1.1+ cells in thymi from PBS- (blue) and KGF-treated mice (red) (n=3-4) were quantified by flow cytometry on day 2 post-treatment. Data represent 3 independent experiments and are shown as mean ± SD. Statistical significance was assessed by unpaired two-tailed Student's t-test: *p<0.05; **p<0.01; ns=not significant.

Figure S4

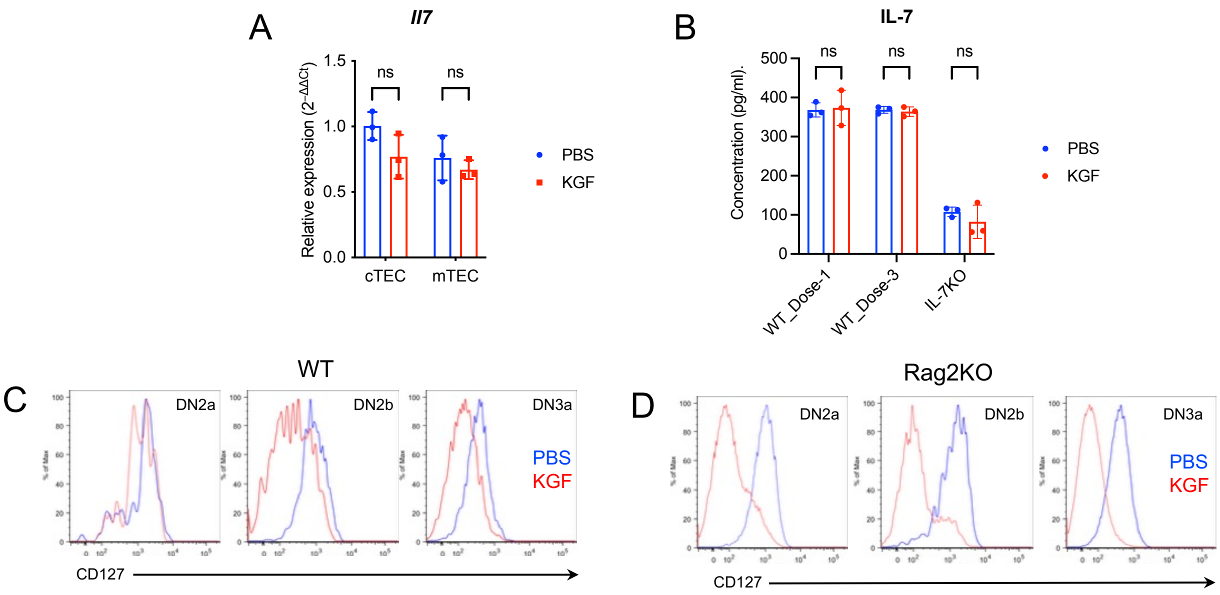

Assessment of IL-7 expression in and IL-7Rα/CD127 expression in DN2a-DN3a thymocytes. (A) Il-7 mRNA levels in cTECs and mTECs from PBS- and KGF-treated mice are presented. (B) Intra-thymic IL-7 protein concentrations were measured in PBS- and KGF-treated mice. Data are shown as mean ± SD from 3 independent experiments. Statistical significance was assessed by unpaired two-tailed Student's t-test. ns=not significant. (C, D) Cell surface expression of CD127 in DN2a, DN2b, and DN3a thymocytes from PBS- (blue) and KGF-treated (red) wild type (C) and Rag2KO (D) mice was evaluated by flow cytometry and representative data from 1 of 3 experiments are shown.

Figure S5

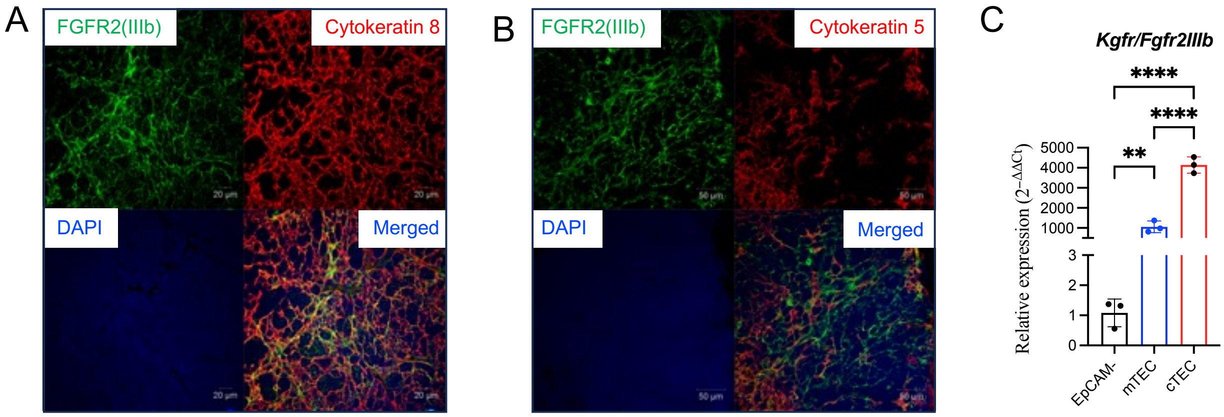

KGFR/FGFR2IIIb expression is restricted to thymic TECs. (A, B) Immunofluorescent staining of frozen thymus sections showing co-localization of KGFR/FGFR2IIIb with cytokeratin 8 (A) and cytokeratin 5 (B) in TECs. (C) *Kgfr*/*Fgfr2IIIb* mRNA levels in sorted mTECs, cTECs, and $EpCAM^{-}UEA^{-}Ly51^{-}$ thymic stromal cells were measured by *TaqMan* gene expression assays. Data represent 3 independent experiments and are shown as mean ± SD. For comparisons among three stromal subsets, one-way ANOVA followed by Tukey's multiple comparisons test was used: **$p<0.01$; ****$p<0.0001$.

Figure S6

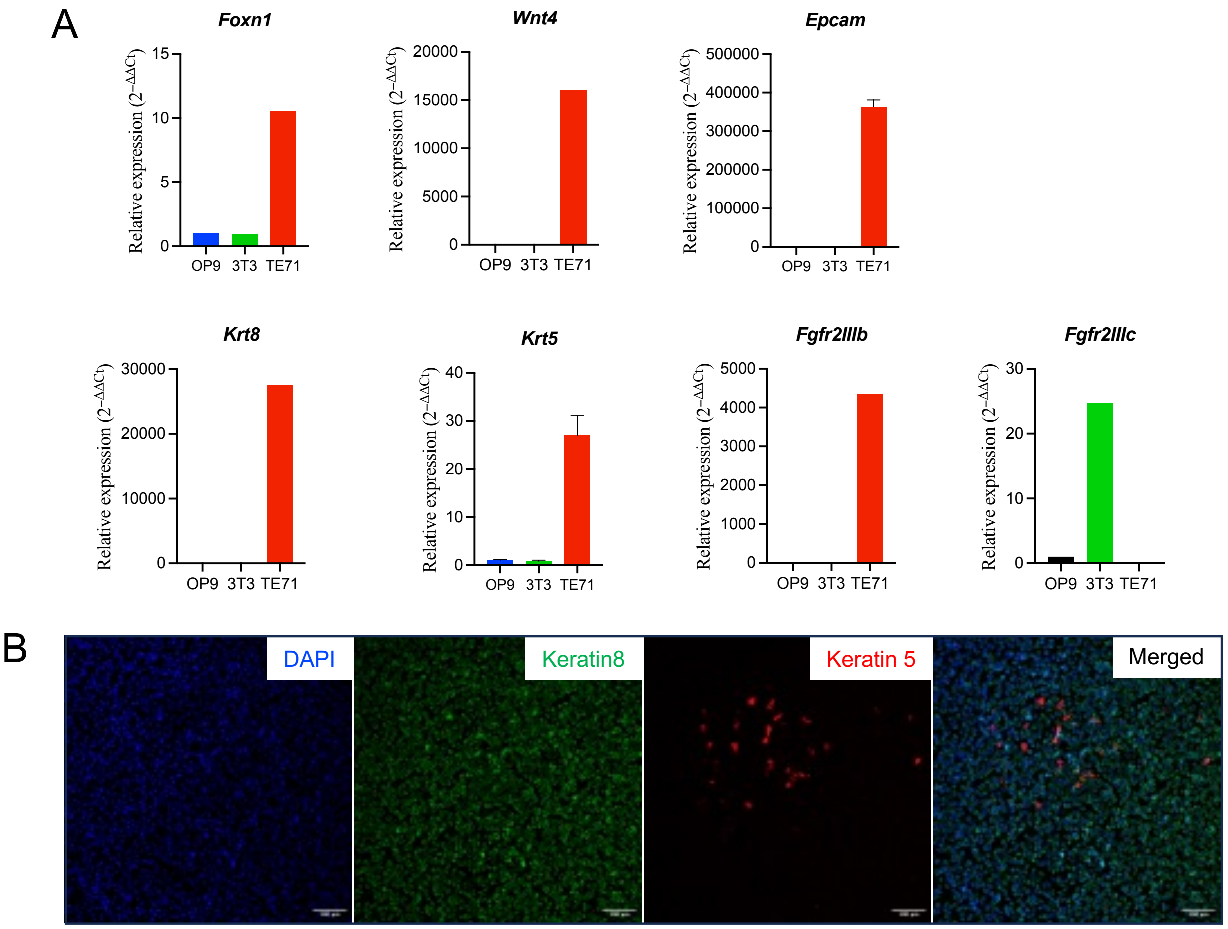

TE-71 cells retain key TEC characteristics. (A) Expression of *Epcam*, *Foxn1*, *Wnt4*, *Krt8*, *Krt5*, *Fgfr2IIIb*, and Fgfr2IIIc were assessed in OP9, 3T3-L1 (3T3), and TE-71 cells by *TaqMan* gene expression assays. (B) Immunofluorescent staining showed cytokeratin 8 expression in the vast majority of TE-71 cells, while cytokeratin 5 was detected in a small subset.

Figure S7

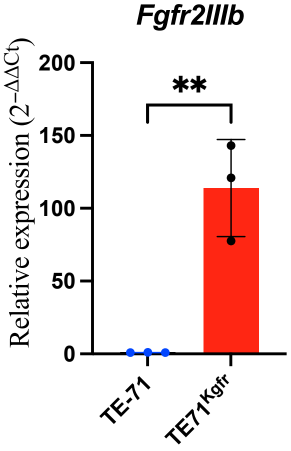

Enhanced expression of *Kgfr/Fgfr2IIIb* in TE-71*kgfr* cells. *Kgfr* expression was evaluated in parental TE-71 cells and *Fgfr2IIIb*-transfected TE-71*kgfr* cells by *Taqman* gene expression assay. Data are presented as mean ± SD (n=3). Statistical significance was determined using an unpaired two-tailed Student's t-test: **p<0.01.

Figure S8

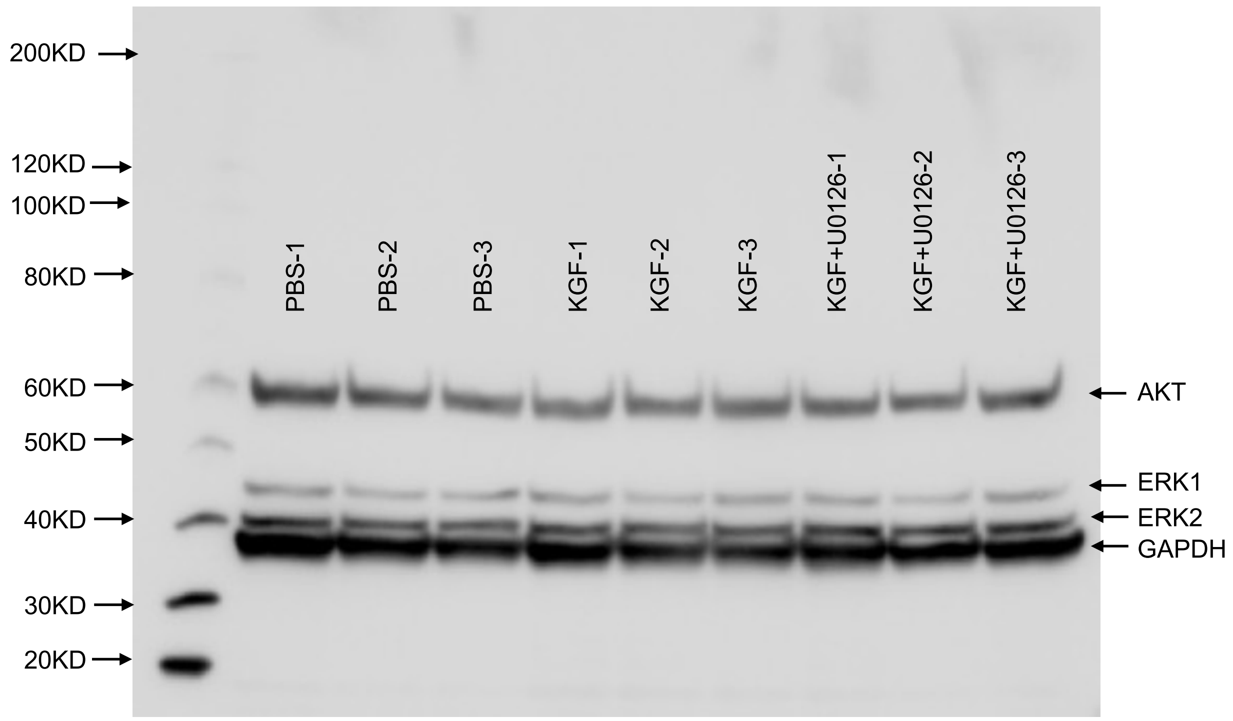

Immunoblot analysis of AKT, ERK1/2, and GAPDH in TE-71^{kgfr} cells following exposure to KGF and U0126. TE-71^{kgfr} cells were cultured for 6 hours in the presence of PBS, KGF, or KGF + U0126 and evaluated for AKT, ERK1/2, and GAPDH expression. This panel shows the original blot corresponding to Figure 7A. Three independent experimental replicates were performed for each condition.

Figure S9

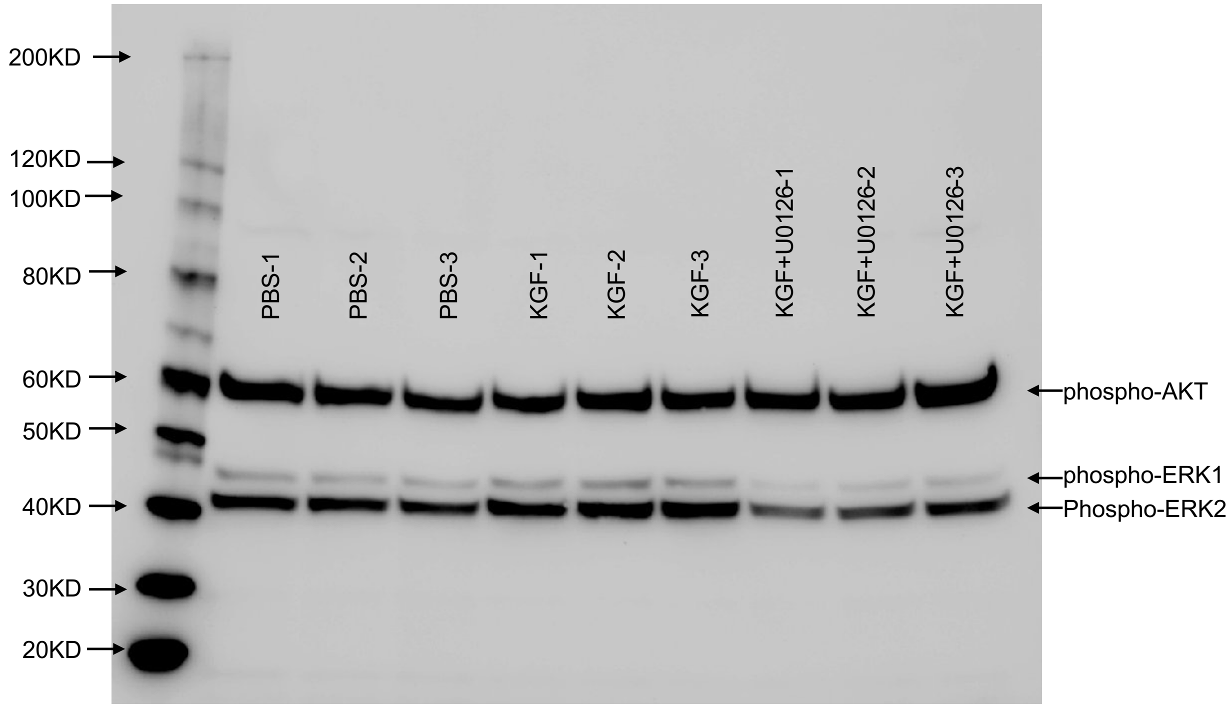

Immunoblot analysis of phospho-AKT and phospho-ERK1/2 in TE-71*kgfr* cells following exposure to KGF and U0126. TE-71*kgfr* cells were cultured for 6 hours in the presence of PBS, KGF, or KGF + U0126 and evaluate for the phosphorylation of AKT and ERK1/2. This panel shows the original blot corresponding to Figure 7A. Three independent experimental replicates were performed for each condition.

Table 1. Antibodies for flow cytometry assays

Number	Product Name	Clone name	Catalog number	Brand name
1	Alexa Fluor 700 anti- mouse CD45	30-F11	103128	Biolegend
2	APC anti-mouse CD326 (EpCAM)	G8.8	118214	Biolegend
3	Brilliant Violet 421 Anti mouse I-A/I-E	M5/114.15.2	107632	Biolegend
4	PE anti-mouse Ly51	6C3	108308	Biolegend
5	Fluorescein Ulex Europaeus Agglutinin I		FL-1061	Vector Laboratories, Inc
6	BD optiBuild BUV395 Rat anti-mouse CD326 (EpCAM)	G8.8	740281	BD Biosciences
7	APC anti-mouse Delta-like 4 (DLL4)	HMD4-1	130814	Biolegend
8	PE anti-mouse Delta-like 4 (DLL4)	HMD4-1	130808	Biolegend
9	Alexa Fluor 700 Rat anti-mouse Ly6G/Ly6c (Gr-1)	RB6-8C5	108417	Biolegend
10	FITC Rat anti-mouseNK1.1	PK136	553164	BD Biosciences
11	Alexa Fluor 488 Rat anti-mouse/human CD11b	M1/70	101217	Biolegend
12	FITC Hamster anti-mouse CD11c	HL3	553801	BD Biosciences
13	FITC anti-mouse CD45R/B220	RA3-6B2	553088	BD Biosciences
14	FITC Rat mAb to Ly76 (TER-119)		561032	BD Biosciences
15	FITC Rat anti-mouse CD4	RM4-4	553055	BD Biosciences
16	FITC anti-mouse CD8a	53-6.7	553031	BD Biosciences
17	Alexa Fluor 488 anti-mouse CD8b	eBIoH35-17.2	53-0083-82	BD Biosciences
18	FITC anti-mouse CD3e	145-2C11	100306	Biolegend
19	APC anti-mouse CD25	PC61	102012	Biolegend
20	APC/Cyanine7 anti-mouse/human CD44	IM7	103028	Biolegend
21	PE Rat anti-mouse Notch1	22E5.5	562754	BD Biosciences
22	PE anti-mouse CD28	E18	122010	Biolegend
23	APC anti-mouse CD28	E18	122010	Biolegend
24	PE anti-mouse CD127 (IL7Ra)	A7R34	135010	Biolegend
25	PE anti-mouse CD117 (c-Kit)	2B8	105808	Biolegend
26	PE anti-mouse TCR γ/δ Antibody	GL3	118108	Biolegend
27	BD Horizon BUV395 anti-mouse CD117 (c-Kit)	2B8	564011	BD Biosciences
28	mouse BD Fc Block™ purified anti-mouse CD16/CD32 mA	2.4G2	553142	BD Biosciences
29	APC Armenian Hamster IgG		400912	Biolegend
30	Purified Mouse Anti-Mouse Pre-T Cell Receptor α Chain	2F5	552407	BD Biosciences
31	PE Armenian Hamster IgG Isotype Ctrl Antibody	HTK888	400907	Biolegend
32	mouse IgG1, κ isotype control	MOPC-31C	557273	BD Biosciences
33	Biotin Rat Anti-Mouse IgG1	A85-1	553441	BD Biosciences
34	mouse anti-mouse FOXN1	2/41		German Cancer Center
35	mouse IgG2b, κ isotype control	MPC-11	557351	BD Biosciences
36	Alexa Fluor™ 647 anti-Mouse IgG (H+L) Antibody	Polyclonal	A32728	Invitrogen

Table 2. List of *Taqman* gene expression assays

Number	Product Name	Catalog number	Brand name
1	Mm00446968_m1 *Hprt*	4331182	Thermo Fisher Scientific
2	Mm00433948_m1 *Foxn-1*	4331182	Thermo Fisher Scientific
3	Mm00487804_m1 *Myc*	4331182	Thermo Fisher Scientific
4	Mm00550265_m1 *Lef1*	4331182	Thermo Fisher Scientific
5	Mm00493445_m1 *Tcf7*	4331182	Thermo Fisher Scientific
6	Mm00484683_m1 *Gata3*	4331182	Thermo Fisher Scientific
7	Mm00432359_m1 *Ccnd1*	4331182	Thermo Fisher Scientific
8	Mm00437341_m1 *Wnt4*	4331182	Thermo Fisher Scientific
9	Mm00437325_m1 *Wnt10a*	4331182	Thermo Fisher Scientific
10	Mm00440330_m1 *Msx1*	4331182	Thermo Fisher Scientific
11	Mm00443610_m1 *Axin2*	4331182	Thermo Fisher Scientific
12	Mm00437347_m1 *Wnt5a*	4331182	Thermo Fisher Scientific
13	Mm00437356_m1 *Wnt7a*	4331182	Thermo Fisher Scientific
14	Mm00444619_m1 *Dll4*	4331182	Thermo Fisher Scientific
15	Mm00432081_m1 *Bmp4*	4331182	Thermo Fisher Scientific
16	Mm00436443_m1 *Ccl25*	4331182	Thermo Fisher Scientific
17	Mm01232337_m1 *Kitl*	4331182	Thermo Fisher Scientific
18	Mm01232339_m1 *Kitl*	4331182	Thermo Fisher Scientific
19	Mm00442972_m1 *Kitl*	4331182	Thermo Fisher Scientific
20	Mm00434291_m1 *Il-7*	4331182	Thermo Fisher Scientific
21	Mm00493214_m1 *Epcam*	4331182	Thermo Fisher Scientific
22	Mm00835759_m1 *Keratin8*	4331182	Thermo Fisher Scientific
23	Mm01305291_m1 *Keratin5*	4331182	Thermo Fisher Scientific
24	Mm00480516_m1 *Bcl11b*	4331182	Thermo Fisher Scientific
25	Mm01213404_m1 *Runx1*	4331182	Thermo Fisher Scientific
26	Mm01342805_m1 *Hes1*	4331182	Thermo Fisher Scientific
27	Mm00492297_m1 *Dtx1*	4331182	Thermo Fisher Scientific
28	Mm00478363_m1 *Ptcra*	4331182	Thermo Fisher Scientific
29	Mm00627185_m1 *Notch1*	4331182	Thermo Fisher Scientific
30	Mm01166193_g1 *Tcrb-J*	4331182	Thermo Fisher Scientific
31	Mm00501300_m1 Rag2	4331182	Thermo Fisher Scientific
32	Mm01340213_m1 *Il2Ra*	4331182	Thermo Fisher Scientific
